# DAF-16/FoxO Directly Regulates an Atypical AMP-Activated Protein Kinase Gamma Isoform to Mediate the Effects of Insulin/IGF-1 Signaling on Aging in *Caenorhabditis elegans*

**DOI:** 10.1371/journal.pgen.1004109

**Published:** 2014-02-06

**Authors:** Jennifer M. A. Tullet, Caroline Araiz, Matthew J. Sanders, Catherine Au, Alexandre Benedetto, Irene Papatheodorou, Emily Clark, Kathrin Schmeisser, Daniel Jones, Eugene F. Schuster, Janet M. Thornton, David Gems

**Affiliations:** 1 Institute of Healthy Ageing, and Department of Genetics, Evolution and Environment, University College London, London, United Kingdom; 2 EMBL, European Bioinformatics Institute, Wellcome Trust Genome Campus, Hinxton, Cambridge, United Kingdom; Stanford University Medical Center, United States of America

## Abstract

The DAF-16/FoxO transcription factor controls growth, metabolism and aging in *Caenorhabditis elegans*. The large number of genes that it regulates has been an obstacle to understanding its function. However, recent analysis of transcript and chromatin profiling implies that DAF-16 regulates relatively few genes directly, and that many of these encode other regulatory proteins. We have investigated the regulation by DAF-16 of genes encoding the AMP-activated protein kinase (AMPK), which has α, β and γ subunits. *C. elegans* has 5 genes encoding putative AMP-binding regulatory γ subunits, *aakg-1-5*. *aakg-4* and *aakg-5* are closely related, atypical isoforms, with orthologs throughout the Chromadorea class of nematodes. We report that ∼75% of total γ subunit mRNA encodes these 2 divergent isoforms, which lack consensus AMP-binding residues, suggesting AMP-independent kinase activity. DAF-16 directly activates expression of *aakg-4*, reduction of which suppresses longevity in *daf-2* insulin/IGF-1 receptor mutants. This implies that an increase in the activity of AMPK containing the AAKG-4 γ subunit caused by direct activation by DAF-16 slows aging in *daf-2* mutants. Knock down of *aakg-4* expression caused a transient decrease in activation of expression in multiple DAF-16 target genes. This, taken together with previous evidence that AMPK promotes DAF-16 activity, implies the action of these two metabolic regulators in a positive feedback loop that accelerates the induction of DAF-16 target gene expression. The AMPK β subunit, *aakb-1*, also proved to be up-regulated by DAF-16, but had no effect on lifespan. These findings reveal key features of the architecture of the gene-regulatory network centered on DAF-16, and raise the possibility that activation of AMP-independent AMPK in nutritionally replete *daf-2* mutant adults slows aging in *C. elegans*. Evidence of activation of AMPK subunits in mammals suggests that such FoxO-AMPK interactions may be evolutionarily conserved.

## Author Summary

Aging is an important problem for human health and is regulated by complex gene regulatory networks. In a simple nematode worm (*Caenorhabditis elegans*) mutation of the insulin/IGF-1 receptor *daf-2* dramatically extends lifespan. This is due to the increased activity of DAF-16, a FoxO transcription factor, leading to altered expression of genes, many encoding other regulatory proteins. We have focused on one such protein, AMP-activated protein kinase (AMPK), that is important for regulating cellular homeostasis under conditions of low energy availability (e.g. starvation). We find that DAF-16 binds to the promoter of *aakg-4* (a gene encoding an atypical γ subunit of AMPK) and increases its expression. Inhibition of *aakg-4* leads to down-regulation of multiple DAF-16 target genes and shortens the life of *daf-2* mutants. Taken together with a previous report showing that AMPK activates DAF-16, this suggests that AAKG-4 and DAF-16 are involved in a positive feedback loop which accelerates effects of DAF-16 on gene expression, and could contribute to longevity. This study defines a new part of the complex gene regulatory network in which DAF-16 acts to control aging. FoxO-AMPK interactions are present in higher animals, where they could potentially also influence aging.

## Introduction

In *Caenorhabditis elegans*, it has long been known that reduction of insulin/IGF-1 signaling (IIS) increases lifespan [1]–[3], reviewed in [4]. However, the mechanisms by which IIS controls aging, and the nature of the aging process itself, remain unclear [5]. One protein that is required for *daf-2* mutants to promote longevity is the FoxO transcription factor DAF-16 [2], [6], [7]. This suggests that DAF-16 regulates expression of terminal effectors of aging. Over the last decade a number of studies have characterized the set of genes regulated by IIS and DAF-16, e.g. [8]–[15]. Although there are many suggestions for how IIS and DAF-16 control aging [9], [16]–[21] how they actually do has proved difficult to determine, particularly because, directly or indirectly, DAF-16 regulates a very large number of other genes.

One approach to investigate DAF-16 function is to define the topology of the gene regulatory network within which it acts. To this end we recently used new chromatin profiling data, cross-referenced to mRNA profile data, to identify with high confidence 65 genes subject to direct transcriptional activation by DAF-16 [22]. The identity of genes in this set gave rise to a new view of DAF-16 action. Rather than acting to regulate a range of somatic maintenance proteins (e.g. detoxification enzymes and chaperonins), DAF-16 targets are involved primarily in signaling and gene regulation, e.g. kinases, phosphatases and transcription factors. This suggests that DAF-16 functions as a central node within a gene regulatory sub-network. Predicted direct DAF-16 target genes include a number of major regulators of metabolism and aging including the AMP-activated protein kinase (AMPK) and, perhaps, DAF-16 itself.

AMPK acts as a fuel gauge within cells: when the AMP∶ATP ratio rises as energy availability drops, activation of AMPK increases catabolism and reduces biosynthesis [23]. Thus, this enzyme helps to coordinate energy availability with rates of biosynthesis and growth. AMPK is heterotrimeric and is formed of an α catalytic subunit and β and γ regulatory subunits. In *C. elegans* there are multiple isoforms of each subunit, *aak-1* and *aak-2* (α), *aakb-1* and *aakb-2* (β), and *aakg-1-5* (γ) (WormBase version WS238). Mammalian AMPK is activated by binding of AMP or ADP to the γ subunit [23], [24].

AMPK inhibits aging in *C. elegans*: The increased lifespan seen in mutants with reduced IIS (e.g. *daf-2*) or TOR (e.g. *rsks-1*) pathways, as well as those with mutations affecting mitochondrial function (e.g. *isp-1*) are *aak-2* (i.e. AMPK) dependent [25]–[27] as is the longevity incurred by a specific form of dietary restriction [28]. Moreover, over-expression or activation of AMPK genes can modestly increase lifespan [25], [28], [29]. Evidence suggests that AMPK may also protect against aging in other species. In *Drosophila* activation of AMPK by modulating enzymes involved in AMP biosynthesis extends lifespan [30] as does a gain-of-function mutation in *lkb1* (an upstream activator of AMPK) [31], although metformin-induced activation of AMPK did not increase lifespan [32]. In mice, mutation of ribosomal S6 kinase (S6K1) increases AMPK activity and slows aging [27], and AMPK activation in response to either AICAR, dietary restriction or physical stimuli declines with age in both rats and mice; reviewed in [33].

That *daf-2* longevity requires *aak-2*, the catalytic subunit of AMPK, may reflect regulation of AMPK by IIS, but the nature of such regulation remains poorly defined [25]. One possibility is that in IIS mutants, DAF-16 promotes longevity by stimulating expression of genes encoding AMPK. Chromatin and mRNA profile data suggest that DAF-16 binds to and activates expression of *aakg-4* and *aakg-5*, and mRNA profile data suggests that DAF-16 also activates expression of *aak-2* and *aakb-1* [22]. Notably, DAF-16 is also phospho-activated by AMPK [28], as is mammalian FoxO3 [34]. This raises the possibility that DAF-16 and AMPK are mutual activators, acting in a positive feedback loop. In this study we have carefully examined the role of DAF-16/FoxO in the regulation of genes encoding AMPK α, β, and γ subunits. We report that the atypical *aakg-4* subunit is a direct target of DAF-16, leading to increased *aakg-4* gene expression. Moreover, reducing *aakg-4* expression attenuates *daf-2* mutant longevity, implying that this regulation contributes to longevity. Effects of *aakg-4* and *daf-2* on expression of DAF-16 target genes support the existence of an AMPK-DAF-16 positive feedback loop promoting diapause and longevity. In addition, the *daf-16* gene itself contains strong DAF-16 binding sites [22], suggesting a possible second positive feedback loop. However, we could find no further evidence that DAF-16/FoxO regulates its own expression.

## Results

### Atypical AMPK γ isoforms in *C. elegans*

Our initial mRNA and chromatin profiling analysis suggested that two AMPK γ subunits, *aakg-4* and *aakg-5*, are direct targets of, and transcriptionally activated by DAF-16 [22]. Binding of AMP to the AMPK γ subunit allosterically activates the enzyme heterotrimer and facilitates phosphorylation of the α subunit by the upstream kinase PAR-4/LKB-1 [23]. *C. elegans* is unusual in possessing five AMPK γ isoforms, compared to one in *Drosophila* [35] and three in humans [36]. Relatively little is known about the function of the five *C. elegans* AMPK γ isoforms but over-expression of a constitutively active form of *aakg-2* (R81Q) can modestly increase worm lifespan [28]. To better understand the function of the five AMPK γ subunits we examined their respective sequences and expression levels.

**Figure 1:**
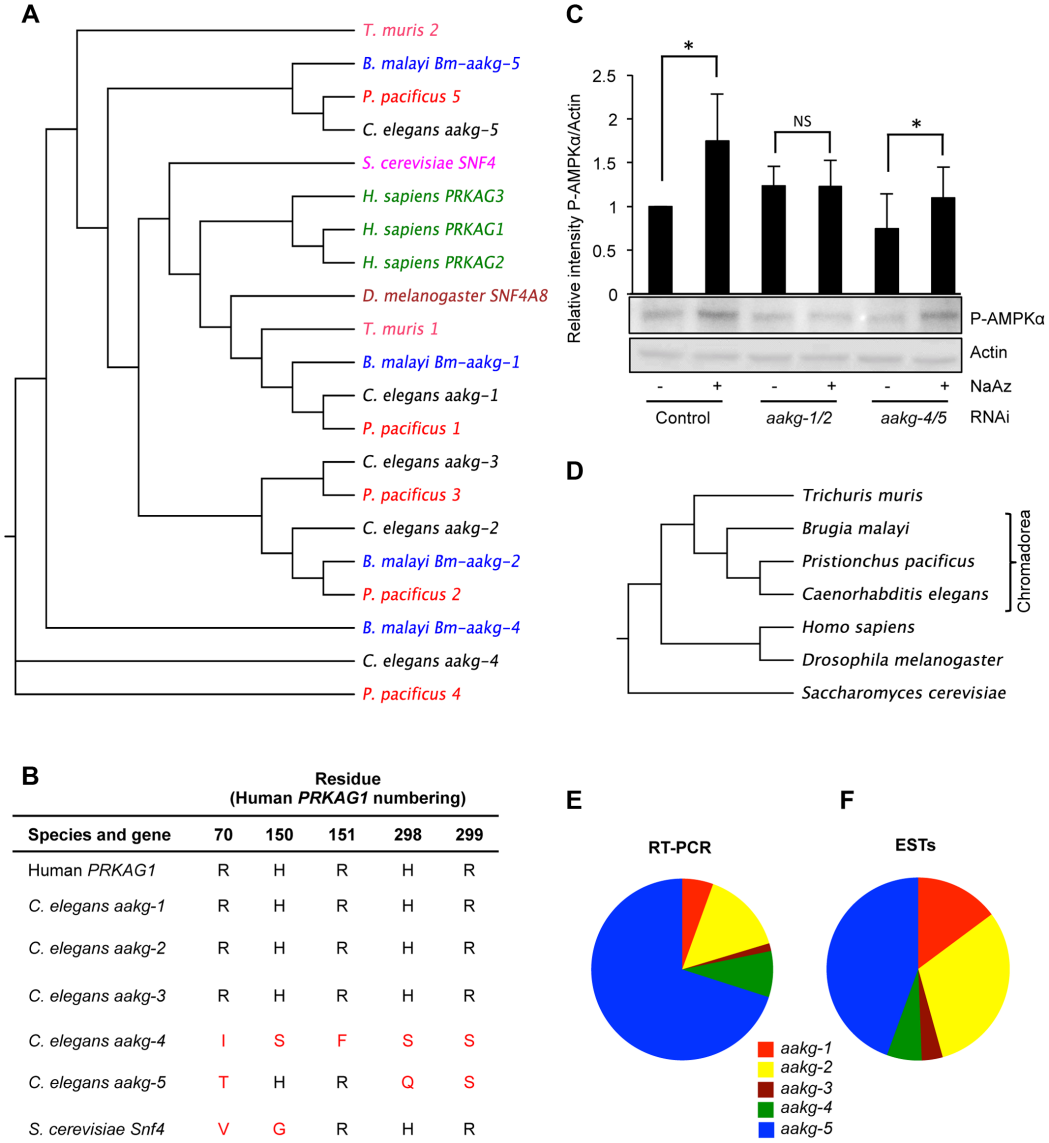
Characterization of five AMPK γ subunits in *C. elegans*. **A**) Phylogenetic relationship between AMPK γ orthologs in selected animal species. **B**) Critical AMP binding residues are missing in yeast *Snf4* and *C. elegans aakg-4* and *aakg-5* (shown in red). An alignment is shown of the human AMPK γ1 (PRAKG1), *C. elegans* γ isoforms (*aakg-1-5*), and budding yeast (*Saccharomyces cerevisiae*) γ, Snf4. Sequences were aligned using the ClusterW multiple alignment application. More broadly, the AMP binding cystathione-β-synthase (CBS) domains [37] are less well conserved in *aakg-4* and *aakg-5* (Table S1). **C**) Evidence that Chromadorian AMPK (containing AAKG-4 or AAKG-5) does not respond to increased AMP levels. Western blot analysis shows levels of phosphorylated AMPKα in wild type (N2) worms in response to 1 mM sodium azide. The assumption was made that *aakg-3* makes little contribution to overall AMPK activity as it is expressed at very low levels (Figures 1E and 1F). Quantification shows means from 4 independent trials. Error bars, standard error. *p<0.05 *vs* control for that RNAi treatment. One representative blot is shown. **D**) Phylogenetic relationship between animal species in 1A. **E, F**) Relative abundance of mRNA of *C. elegans* AMPK γ isoforms based on **E**) quantitative qRT-PCR and **F**) numbers of ESTs (WormBase version WS237).

In terms of sequence similarity to mammalian AMPK γ isoforms, *aakg-1* is the most similar, followed by *aakg-2* and *aakg-3* (Figure 1A and Figures S1 and S2). The remaining isoforms, *aakg-4* and *aakg-5*, are more divergent and form a distinct clade, suggesting that they resulted from a duplication of an ancestral atypical AMPK γ isoform. The sequence alignment of the *C. elegans* γ isoforms with the human AMPK γ1 (PRKAG1) isoform highlights conserved regions corresponding to the mammalian AMPK cystathione-β-synthase (CBS) domains. These domains mediate AMP/ADP and ATP binding to the enzyme allowing AMPK to respond to changes in the energy status of the cell. Interestingly, there is much less CBS domain conservation in *aakg-4* and *aakg-5* (Table S1) and some key residues required for interaction with AMP/ADP/ATP are missing: residues Arg-70 (human PRKAG1 numbering); His-150/Arg-151; and His-298/Arg-299 (Figure 1B) [37]. Their importance is highlighted by the fact that mutation of some of these residues in the heart-specific human AMPK γ2 isoform (R302Q, H383R, R384T, R531G/Q) have been identified in humans suffering from Wolff-Parkinson-White syndrome with cardiac hypertrophy and glycogen storage disease [38]–[41]. Studies have shown that these mutations lead to impaired activation by AMP/ADP [24], [42], [43]. Thus, AMPK complexes harboring any of these mutations no longer respond to stimuli that alter the AMP/ADP∶ATP ratio [44]. This suggests that *C. elegans* AMPK complexes containing AAKG-4 or AAKG-5 might render it AMP/ADP insensitive.

To probe whether AMPK containing AAKG-4 or AAKG-5 might be AMP/ADP insensitive, we compared worms subjected to simultaneous *aakg-4/5*(RNAi), in which one would expect all AMPK to be AMP/ADP sensitive, with *aakg-1/2*(RNAi) worms in which one would expect most AMPK to be AMP/ADP insensitive. To test AMPK activity, we measured levels of AMPKα phosphorylation, either in control, nutritionally replete animals, or in ATP-depleted animals (treated with the respiratory inhibitor sodium azide). As predicted, RNAi control animals treated with azide showed increased AMPK activity (Figure 1C). Our expectation was that this increase was mediated by AMPK containing AAKG-1 or AAKG-2. Consistent with this, azide increased AMPK activity in *aakg-4/5*(RNAi) worms but not *aakg-1/2*(RNAi) worms (Figure 1C). In nutritionally replete animals, presumably with lower AMP levels, one would expect that most AMPK activity was from enzyme containing AAKG-4 or AAKG-5. Consistent with this, we observed a slight decrease in AMPK activity in response to *aakg-4/5*(RNAi) but not *aakg-1/2*(RNAi) (Figure 1C). These results support the hypothesis that the activity of AMPK containing AAKG-4 or AAKG-5 is constitutively active, and not AMP dependent. However, to be certain of this, further studies would be needed, e.g. of the properties of reconstituted *C. elegans* AMPK *in vitro* using recombinant proteins.

### Evolution of Chromadorean AMPK γ isoforms

To investigate the evolution of the five *C. elegans aakg* subunits, and the extent of the existence of atypical AAKG isoforms in other species, we identified and analyzed AAKG protein sequences from a range of nematode species. Orthologs of *aakg-4* and *aakg-5* were found in the nematodes *Pristionchus pacificus* (order: Rhabditida) and *Brugia malayi* (order: Spirurida) (Figures 1A, and 1D), demonstrating that these two isoforms are widespread at least among the large Chromadorea class of nematodes. However, orthologs of AAKG-4 and -5 are not clearly identifiable among either the more distantly related nematodes of the class Enoplea (e.g. *Trichuris muris*), or among platyhelminth species (Figures S1 and S3). This phylogenetic analysis suggests that duplications separating *aakg-4* from *aakg-5* and *aakg-1* from *aakg-2,-3* occurred before the common ancestor of the Rhabditida and Spirurida (Figure 1D). The final duplication separating *aakg-2* from *aakg-3* appears to have happened later, prior to the ancestor of the genera *Caenorhabditis* and *Pristionchus* as most of the Rhabditid species contain five *aakg* paralogs (Figure S2), but Spirurid nematodes such as *B. malayi* possess an ortholog of *aakg-2* but not *aakg-3* (Figure S1).

Notably, all orthologs of *C. elegans aakg-4* and *-5* also lack key AMP-binding residues (Tables S2 and S3), implying that the single ancestor of these two genes also lacked these residues. Some AAKG isoforms in the more distantly related Enoplea nematodes also lack key AMP binding residues (Figure 1A, Figure S1 and Table S3), raising the possibility that atypical AAKG isoforms might have existed in the common ancestor of the Enoplea and the Chromadorea.

In order to gain some indication of the relative importance of the five AMPK γ isoforms in *C. elegans*, we measured levels of their respective mRNAs in wild-type adult hermaphrodites. The most abundant mRNA was that of the atypical isoform *aakg-5* (70.1% of total *aakg* mRNA) (Figure 1E), while the other atypical isoform *aakg-4* accounted for 8.2% of *aakg* mRNA (Figure 1E). A count of the number of ESTs for each isoform listed on WormBase confirmed that *aakg-5* is the most abundant mRNA (Figure 1F). Thus, the two atypical isoforms account for more than 3/4 of the total *aakg* mRNA. This implies that that much of the AMPK trimer in adult hermaphrodites contains either AAKG-4 or AAKG-5. For convenience, we suggest the term Chromadorean AMP kinase to describe this unusual form of this major metabolic regulator.

### IIS regulation of atypical AMPK isoforms

**Figure 2:**
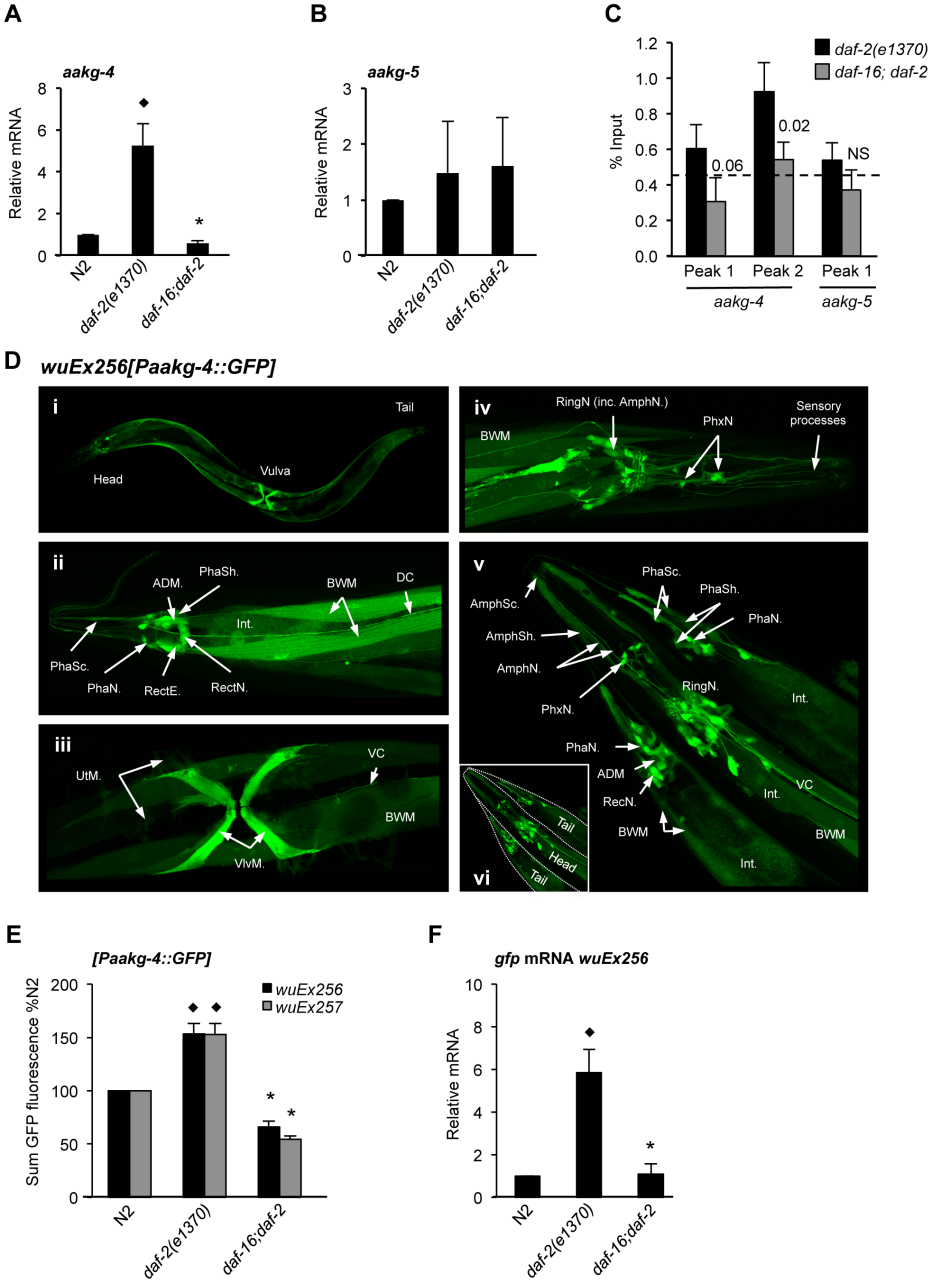
*aakg-4* transcription is directly regulated by DAF-16/FoxO. **A, B**) *aakg-4* but not *aakg-5* mRNA levels are increased in *daf-2* animals in a *daf-16* dependent manner. ^♦^ *p*<0.05, compared to N2, *p<0.05, compared to *daf-2*. Mean values from 4 trials. Error bars, standard deviation. **C**) DAF-16 binds to the promoter of *aakg-4* but not *aakg-5*. *aakg-4* peak 2 is positioned within 1 Kb of the transcriptional start site and contains two DAF-16 binding elements (Figure S5). One representative experiment is shown which contained 3 immuno-precipitation replicates from the same chromatin preparation (error bars show the standard deviation between them). The horizontal dotted line shows the % input from a negative control region (3′ of gene R11A5.4) that does not bind DAF-16 [22]. Statistical analysis of additional trials is presented in Table S5. A western blot showing the specificity of the DAF-16 antibody is shown in Figure S4. **D**) Confocal microscopy shows *Paakg-4::gfp* to be broadly expressed in *C. elegans*. Images show *Paakg-4::gfp* expression pattern in 1 day old hermaphrodites. (**i**) Whole worm expression pattern. (**ii**) *Paakg-4::gfp* is seen in tail sensory organs: phasmid sheath (PhaSh), socket cells (PhaSc) and neurons (PhaN), as well as in epithelial rectal cells (RectE), anal-depressor muscle (ADM), pre-anal ganglion rectal neurons (RectN), body wall muscles (BWM), posterior intestine (Int) and dorsal cord neuronal processes (DC). (**iii**) *Paakg-4::gfp* is expressed in vulval (VlvM), uterine muscles (UtM) and ventral cord processes (VC). (**iv**) Head expression mostly localizes to the ring ganglia (RingN) plus 6 pharyngeal neurons (PhxN). (**v**) It is also seen in amphid sensory organs including sheath (AmphSh), socket cells (AmphSc) and neurons (AmphN). Table S10 compares expression of *Paakg-4::gfp* with other AMPK subunits. **E**) Quantification of GFP fluorescence in worms expressing the *Paakg-4::gfp* reporter shows that fluorescence increased in *daf-2* animals dependent on *daf-16*. The same was also true for a second set of strains generated from a different extrachromosomal array. Means from 3 independent trials shown. Error bars, standard error. Animals contained the *wuEx256* transgene array. ^♦^ *p*<0.01 compared to N2, *p<0.001 compared to *daf-2*. **F**) *gfp* mRNA was increased in *daf-2* animals in a *daf-16*-dependent fashion. ^♦^ *p*<0.05 compared to N2, *p<0.01 compared to *daf-2*. Means from 3 independent trials shown. Error bars, standard deviation. Prior to transgene quantification animals were maintained at 15°C and then shifted to 25°C for 24 hr at the L4 stage.

Next we examined regulation by IIS and DAF-16 of genes encoding AMPK γ subunits. A previous transcript profile analysis showed 6.6-fold and 3.5-fold (both *p*<0.001) increases in *aakg-4* and *aakg-5* mRNA, respectively, in *glp-4(bn2); daf-2(m577)* relative to *daf-16(mgDf50) glp-4; daf-2* young adult hermaphrodites [8]. Further data mining showed similar results for *aakg-4* in an additional two profiles [15], [45] and for *aakg-5* in one profile [15] (Table S4). For verification, we used qRT-PCR to compare mRNA levels in *daf-2(e1370)* and *daf-16; daf-2* young adults. This confirmed that *aakg-4* mRNA levels are elevated in *daf-2* relative to *daf-16; daf-2*, but for *aakg-5* this could not be confirmed (Figure 2A, 2B and Table S4).

Next we verified binding of DAF-16 to the promoters of *aakg-4* and *aakg-5*, comparing *daf-2(e1370)* and *daf-16; daf-2* adults using chromatin immunoprecipitation (ChIP) with a DAF-16-specific antibody (Santa Cruz) (Figure S4) and PCR. Our initial chromatin profiling experiment [22] had identified two DAF-16 binding peaks associated with *aakg-4* and one with *aakg-5* (Figure S5) and we examined each individually. We confirmed DAF-16 binding in the *aakg-4* peak situated within 1 Kb of the transcriptional start site (peak 2) (Figure 2C and Table S5). However, we did not consistently observe DAF-16 binding at the first *aakg-4* peak or that of *aakg-5* (Figure 2C and Table S5). Thus, it seems that *aakg-4* is a *bona fide* DAF-16 target gene and DAF-16 binding to the *aakg-5* promoter is either non-existent or condition dependent.

To examine further the expression of *aakg-4* and *aakg-5*, and their regulation by IIS and DAF-16, we created transgenic lines containing *Paakg-4::gfp* and *Paakg-5::gfp* transcriptional reporters. *Paakg-4::gfp* expression was observed in many tissues, particularly the intestine, body wall muscle, vulval muscles and several neurons in the head and tail including amphids and phasmids, respectively (Figure 2D). A broadly similar pattern of expression was seen in all larval stages and adults (data not shown). *Paakg-5::gfp* was also widely expressed (Figure S6A) and although expression of the two isoforms overlapped to an extent, there were also clear differences. For instance, *aakg-5* was not expressed in amphid neurons nor *aakg-4* in the pharynx (Figure 2D and Figure S6A).

To further assess the impact of IIS on *aakg-4* and *aakg-5* expression, transgene arrays were crossed into *daf-2(e1370)* and *daf-16; daf-2* backgrounds and GFP fluorescence measured. Consistent with *aakg-4* mRNA data, *Paakg-4::gfp* expression was increased 1.6 fold by *daf-2*, and this increase was *daf-16* dependent (Figure 2E). No changes were detected in the distribution of expression (data not shown).

It was recently shown that mutation of *daf-2* globally reduces protein levels by inhibiting protein synthesis [46], [47] raising the concern that GFP levels in *daf-2; Paakg::gfp* worms might be affected by non-specific effects of *daf-2* on protein synthesis. We therefore introduced an additional control, using qRT-PCR to measure effects of IIS on *gfp* mRNA levels. In *Paakg-4::gfp* transgenic worms this revealed a greater, 6-fold induction in expression of *gfp* mRNA by *daf-2* (Figure 2F). In contrast, no effect of IIS was detected on the level of *Paakg-5::gfp* GFP fluorescence, *gfp* mRNA or on distribution of expression (Figures S6B, S6C and data not shown).

We also examined the *aakg-4* promoter sequence, which proved to contain two predicted DAF-16-binding elements (DBE) [48] (POSSUM scores 9.18 and 10.1), one of which is canonical and within 1 Kb of the translational start site (Figure S5). This is within the DAF-16-binding site previously identified by chromatin profiling using DamID and confirmed by ChIP PCR [22] (Figure 2C). One canonical DBE (POSSUM score 9.06) is also present in the same region of the *C. briggsae aakg-4* promoter, suggesting conservation of regulation by IIS/FoxO, but not in *C. remanei*. DBEs were not detected in the *aakg-5* promoter of any of these species. These findings strongly imply that DAF-16 binds to the promoter of *aakg-4* and activates its expression.

### *aak-2*, *aakb-1* and IIS/DAF-16 regulation

We also checked our previous whole genome mRNA profiling study comparing *glp-4; daf-2(m577)* relative to *daf-16; glp-4; daf-2* young adult hermaphrodites [8] for changes in expression of other AMPK subunits. *aak-1* (α1 subunit) and *aakb-2* (β2) mRNA levels were not different between the two strains, but *aak-2* (α2) and *aakb-1* (β1) were both elevated in *daf-2 vs daf-16; daf-2*, showing 2.28-fold and 7.51-fold (both *p*<0.001) higher levels, respectively, in *daf-2*. *aakb-1* activation by DAF-16 was also implied by two other mRNA profile studies [15], [45], although not by a third study [49]. *aakb-1* mRNA levels were also higher in *daf-2(e1370)* relative to N2 in individually profiled 19 day old worms [50] (Table S4). Changes in *aak-2* mRNA levels were not seen in any of these other studies. For verification, we then used qRT-PCR to compare *aak-2* and *aakb-1* mRNA levels in 1 day old *daf-2(e1370)* and *daf-16; daf-2* adults. This confirmed a statistically significant increase for *aakb-1*, but also suggested a possible increase in *aak-2* mRNA levels (Figure S7 and Table S4). These results demonstrate that DAF-16 robustly increases *aakb-1* expression, and suggest that it might also activate *aak-2* expression. We also compared expression of *Paak-2::gfp* and *Paakb-1::gfp* transcriptional reporters [51] (Figures S8A and S9A) in wild type, *daf-2* and *daf-16; daf-2* backgrounds. For neither reporter was any effect of IIS on expression detected, either in terms of level (Figures S8B and S9B) or distribution (data not shown). However, in the case of *Paak-2::gfp* levels of *gfp* mRNA were higher in *daf-2* relative to *daf-16; daf-2* (Figures S8C), and possibly also for *Paakb-1::gfp* (Figures S9C). Thus, there appears to be a modest *daf-16*-dependent increase in both *aak-2* and *aakb-1* expression in *daf-2* mutants, but one which is, perhaps, condition dependent and near the borderline of detectability.

The evidence of up-regulation of *aakb-1* expression by DAF-16 suggests that this AMPK subunit might play a role in *daf-2* mutant longevity (the Age phenotype). To test this the deletion mutation *aakb-1(tm2658)* was combined with *daf-2(m577)*, and lifespan measured at 25°C. *aakb-1* proved to shorten lifespan in *daf-2(m577)* and wild type worms to a similar degree (12% and 11%, respectively; mean of 4 trials) (Figure S10 and Table S6). To estimate whether the effect of *aakb-1* differed between wild type and *daf-2* backgrounds we used Cox proportional hazard analysis (CPHA) on combined data for all trials, but no difference was detected (*p* = 0.25). This suggests that activation of *aakb-1* expression does not contribute to *daf-2* Age.

### *aakg-4* contributes to the *daf-2* Age phenotype

**Figure 3:**
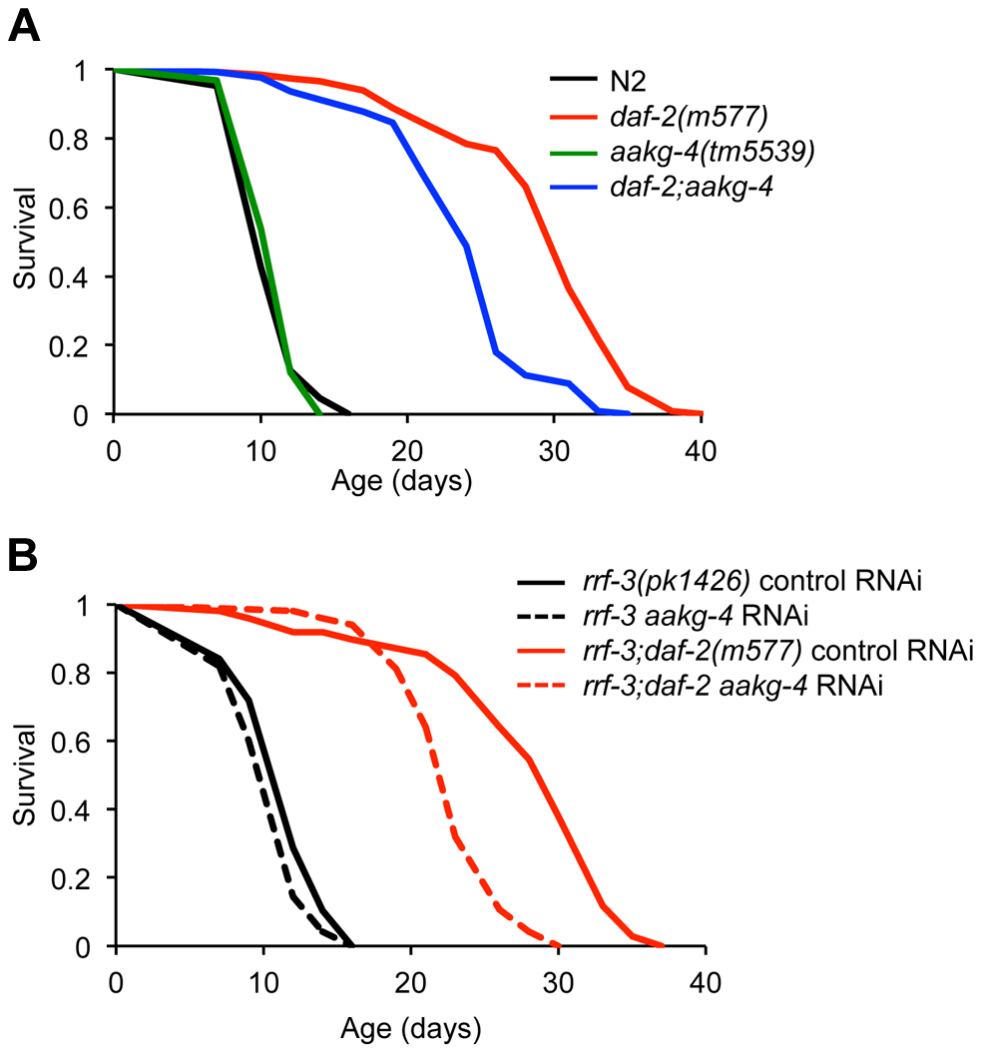
*aakg-4* mutation or *aakg-4*(RNAi) shorten the lifespan of *daf-2(m577)* animals. **A and B**) One representative experiment is shown in each case, corresponding to **A**) Trial 1 in Table S7 and **B**) Trial 5 in Table S8. Lifespan measurements carried out at 25°C.

To assess whether *aakg-4* contributes to *daf-2* Age, we obtained the mutation *aakg-4(tm5539)*. This deletes 405 bp from the gene, in a region encoding portions of both CBS3 and CBS4, and is therefore predicted to be a null. *aakg-4* mutants appeared healthy and showed normal fertility (Figure S11). *aakg-4* reduced lifespan in a *daf-2(m577)* background (−24.4%, combined data for 4 trials), but not in a *daf-2(+)* background (Figure 3A, Table 1 and Table S7). Statistical analysis confirmed that *aakg-4* effects on lifespan were greater in a *daf-2* background (CPHA *p*<0.0001), implying that *aakg-4* contributes to *daf-2* Age. To confirm this, we abrogated *aakg-4* expression using RNAi, and again observed a greater reduction in lifespan in *daf-2* than in wild-type worms (9 trials, CPHA *p* = 0.0024) (Figure 3B, Table 1 and Table S8).

**Table 1:** *aakg-4(tm5539)* and *aakg-4* RNAi shortens *daf-2* lifespan.

Strain	Genotype	Mean lifespan	% Difference	N (total)	*p*
	N2	11.5		493 (499)	-
GA1071	*aakg-4(tm5539)*	11.8	+2.96^b^	212 (246)	<0.0001^a^, NS^b^
DR1567	*daf-2(m577)*	29.4		442 (448)	<0.0001^b^
GA1072	*daf-2(m577);aakg-4(tm5539)*	23.5	−25.09^a^	661 (665)	<0.0001^a,b,c^
NL2099	*rrf-3(pk1426)* control RNAi	11.7		603 (665)	-
NL2099	*rrf-3 aakg-4* RNAi	10.6	−9.43^b^	493 (532)	<0.0001^b^
NL2099	*rrf-3 aakg-5* RNAi	10.9	−6.33^b^	645 (689)	NS^b^
GA303	*rrf-3; daf-2* control RNAi	26.3		673 (704)	<0.0001^b^
GA303	*rrf-3; daf-2 aakg-4* RNAi	22.5	−14.34^a^	403 (403)	<0.0001^a,b^
GA303	*rrf-3; daf-2 aakg-5* RNAi	22.7	−13.89^a^	209 (216)	<0.0001^a,b^

Table shows combined data from 4 *aakg-4(tm5539)* trials and 9 *aakg-4/aakg-5* RNAi trials; for individual trials see Tables S7 and S8.

% difference in mean lifespan compared to ^a^ *daf-2(m577)* or ^b^ N2.

N, number of worm deaths (total worms scored including those censored).

*p*, probability (determined by log rank test) of being the same as specified control: ^a^ *daf-2(m577)*, ^b^ N2 or ^c^ *aakg-4(tm5539)*. NS, not statistically significant (*p*>0.05).

To test whether *aakg-5* also contributed to *daf-2* Age we used *aakg-5*(RNAi). Like *aakg-4*(RNAi), this too markedly reduced lifespan in *daf-2* but not N2 (4 out of 9 trials, CPHA, *p* = 0.0005) (Figure S12 and Table S8). We also tested effects of RNAi of *aakg-1*, *aakg-2* and *aakg-3* but none consistently suppressed *daf-2* Age (Table S9).

*aakg-4* and *aakg-5* form a distinct clade within the *C. elegans* AMPK γ subunits (Figure 1) and both contribute to *daf-2* Age. This raises the possibility is that these two genes are partially redundant in function. To probe this we tested whether *aakg-5*(RNAi) could further reduce *daf-2* Age in *daf-2;aakg-4* worms, but it could not (Figure S12 and Table S8), suggesting that *aakg-4* and *aakg-5* are not functionally redundant.

Taken together, these findings imply that in *daf-2* mutants, activation of DAF-16 leads to increased *aakg-4* expression, which in turn contributes to the *daf-2* Age phenotype. *aakg-5* may also be important for *daf-2* Age but is not directly regulated by DAF-16. The absence of key residues important for interacting with AMP in AAKG-4 and our data implying that AAKG-4 and AAKG-5 activity may be unresponsive to AMP/ADP (Figures 1B and 1C) suggests the possibility that *daf-2* mutants have elevated expression of an AMP-independent AMPK complex (Chromadorean AMPK).

### Evidence of a positive feedback loop involving DAF-16 and AMPK

We have identified *daf-16*-dependent up-regulation of expression of *aakg-4*, and possibly also *aak-2* and *aakb-1*, in *daf-2* mutants. It was shown previously that AMPK directly phosphorylates and activates DAF-16 [28]. Taken together, this suggests the presence of a positive feedback loop, where DAF-16 increases AMPK activity by promoting expression of genes encoding AMPK subunits, and this in turn activates DAF-16 activity. If such a positive feedback loop exists, then *aakg-4* should promote DAF-16 activity. To test this we used expression of DAF-16 target genes as a readout of DAF-16 activity.

**Figure 4:**
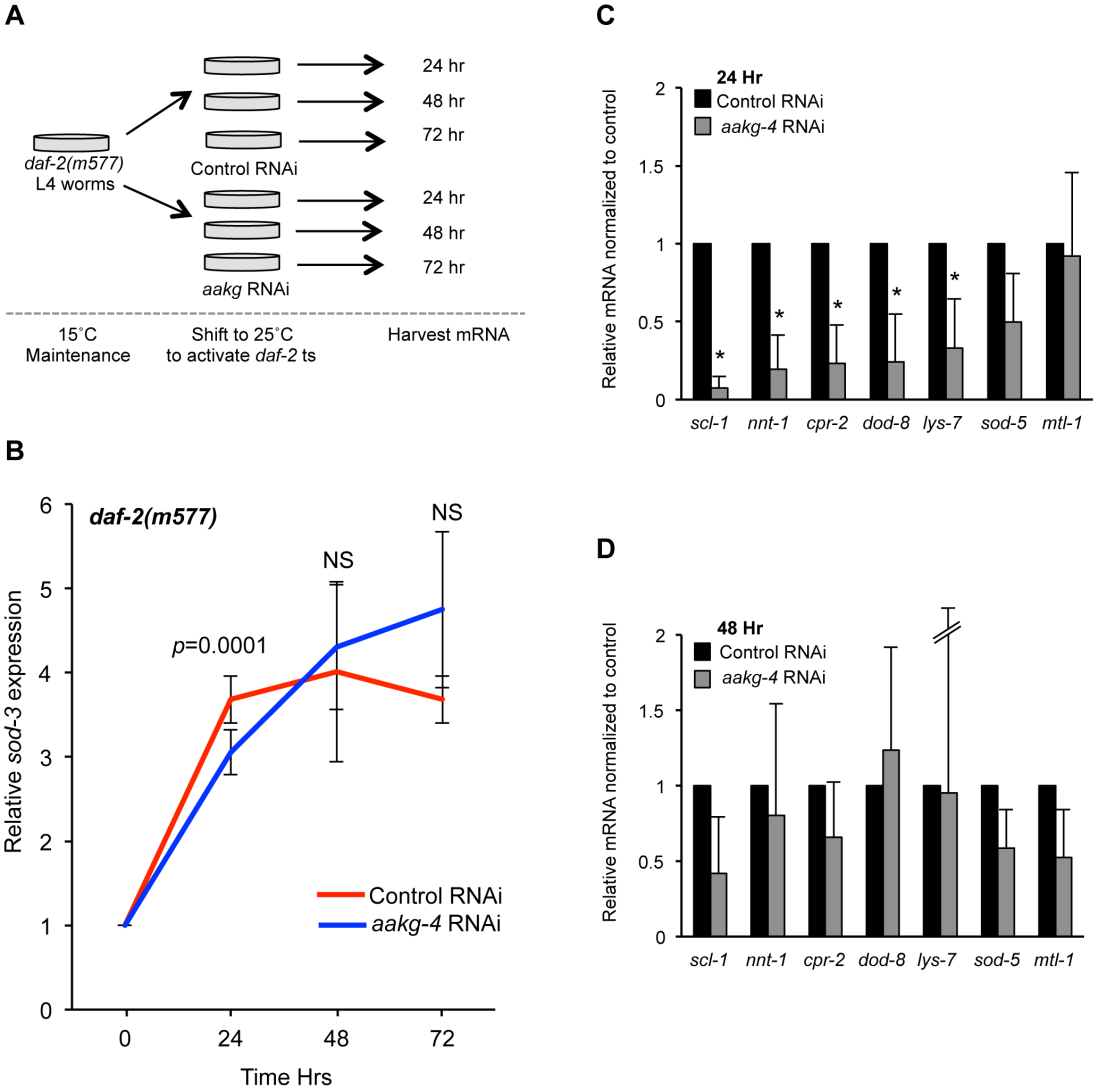
*aakg-4* RNAi alters dynamics of DAF-16 target gene expression. **A**) Experimental design: L4 *daf-2(m577)* animals were treated with either control (L4440) or *aakg-4* RNAi and shifted to 25°C prior to RNA extraction. **B**) The induction of *sod-3* mRNA expression is reduced after 24 hr by *aakg-4*(RNAi) but this reduction was not seen after 48 or 72 hr. The level of *sod-3* at 0 hr is that of adults that have not been shifted to 25°C (i.e. maintained at 15°C). **C, D**) The expression of other DAF-16 target genes was also reduced after 24 hr by *aakg-4*(RNAi) but this reduction was not seen after 48 hr. Error bars, standard deviation. *p<0.05 compared to *daf-2* animals fed control RNAi.

Expression of the well-characterized DAF-16 target gene *sod-3* is induced by reduced IIS and has also been shown to be regulated by *aak-2* [28]. *sod-3* expression can be induced by shifting *rrf-3; daf-2(m577*) worms from 15°C to 25°C (the non-permissive temperature for this temperature-sensitive *daf-2* allele) (Figure S13). The *rrf-3* mutation sensitizes the animals to RNAi [52]. A temperature shift does not induce *sod-3* expression in either *rrf-3* or *rrf-3; daf-16; daf-2* animals (Figure S13). Notably, if *aakg-4*(RNAi) was initiated at the same time as the temperature shift there was a significant reduction in *sod-3* expression after 24 hr in animals treated with *aakg-4*(RNAi) (Figure 4A and 4B). Moreover, this reduction was transient, and absent at later time points (48 hr or 72 hr).

We then looked at the effect of *aakg-4*(RNAi) in *rrf-3; daf-2(m577*) worms on expression of seven other known DAF-16 target genes [53], [54] 24 hr or 48 hr after temperature shift/RNAi and found that 5 out of 7 also showed reduced expression compared with control RNAi after 24 hr but, again, not 48 hr (Figure 4C and 4D). Next, we tested the effects of *aakg-1*, *aakg-2* and *aakg-5* RNAi on *sod-3* expression but could detect no significant effect (Figure S14), implying that DAF-16 activity is specifically dependent on *aakg-4*. Finally, we tested whether *aakg*(RNAi) in *daf-2* mutant animals reduced nuclear localization of DAF-16::GFP, but only marginal effects were detected (Figure S15). This is consistent with a previous report that phosphorylation of DAF-16 by AMPK does not increase nuclear localization [28].

**Figure 5:**
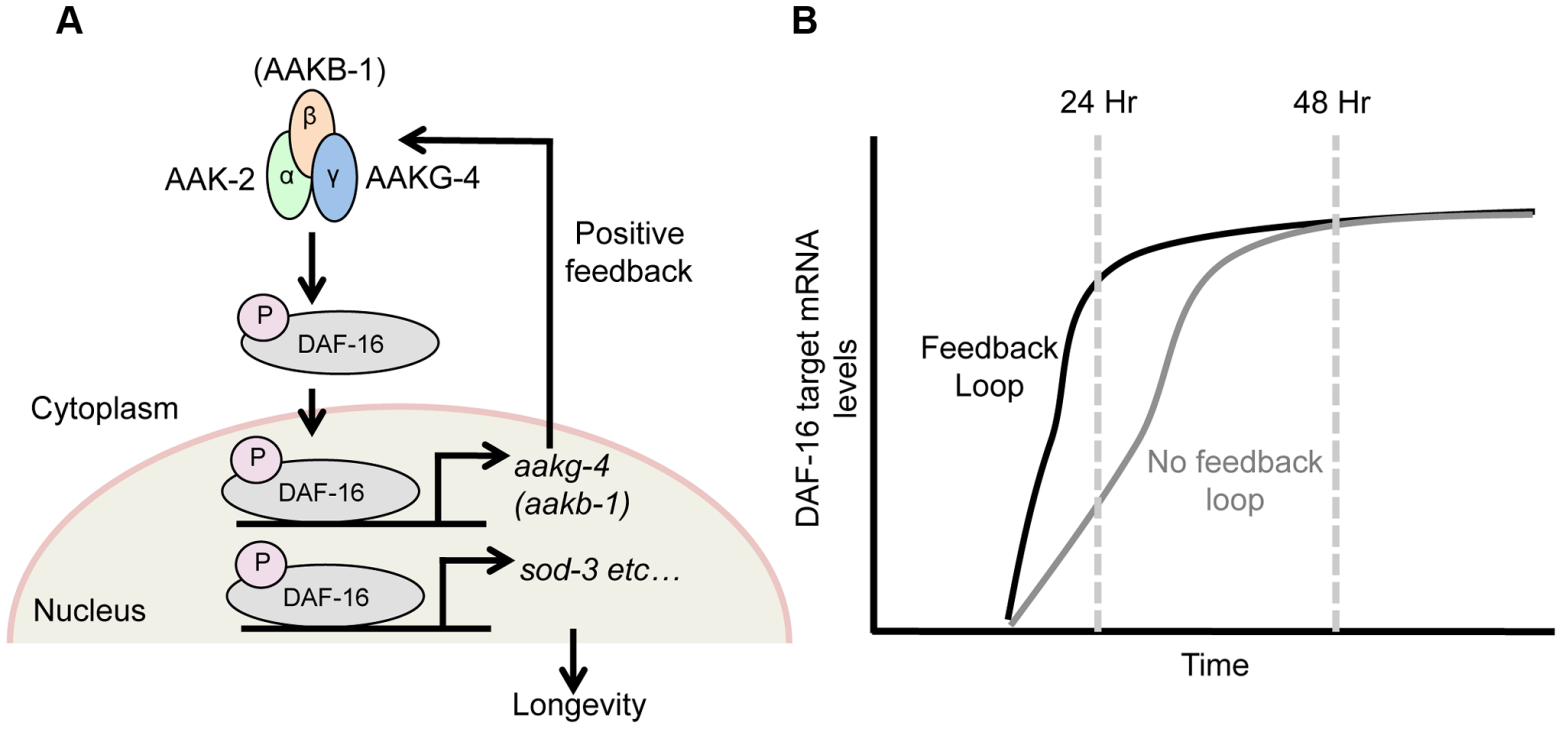
DAF-16 and AMPK form a positive feedback loop. Schematic model suggested by combining findings presented here, and elsewhere [28]. **A**) AMPK phospho-activates DAF-16 [28] and directly controls the transcription of 2 AMPK subunits, *aakg-4* and possibly *aakb-1* (this study). This in turn further increases AMPK levels, which further activate DAF-16 and target gene expression, and so on, creating a positive feedback loop. This broadly affects expression of DAF-16 target genes (e.g. *sod-3*), including those that determine aging rate (though note that *sod-3* itself does not affect aging [77], [78]. **B**) This feedback loop appears to speed up DAF-16-mediated induction of gene expression in response to reduced IIS, rather than altering final expression level.

These results imply that AAKG-4 can increase DAF-16 activity, consistent with the existence of a positive feedback loop involving Chromadorean AMPK (Figure 5A). That reduced induction of DAF-16 target gene expression is seen after 24 hr but not 48 hr suggests that the function of the positive feedback loop is to accelerate DAF-16 target gene induction, rather than to alter their endpoint expression levels (Figure 5B).

### *daf-16* does not activate its own expression

Chromatin profiling data [22] implies that DAF-16 binds strongly to its own promoter, suggesting the possible action of DAF-16 in a second positive feedback loop involving activation of its own transcription. To test whether *daf-16* activates its own transcription, we compared *daf-16* mRNA levels in *daf-2(e1370)* mutants in the presence of the *daf-16(m26)* point mutation. No difference was detected, implying that *daf-16* does not activate its own expression (Figure S16A). This conclusion was further supported by studies of *Pdaf-16a::gfp* reporters (Figures S16B–F).

## Discussion

In this study, we used existing mRNA and chromatin profiling data as a starting point for careful testing of predicted gene regulatory relationships involving DAF-16. This has revealed the presence of a positive feedback loop involving DAF-16 and AMPK, where DAF-16 activation of an atypical AMPK γ subunit plays a key role.

### *aakg-4* as an effector of DAF-16/FoxO

Our results show that DAF-16 acts directly on the promoter of *aakg-4* to activate its transcription. The observation that *daf-2* mutants have elevated *aakg-4* levels suggested that this may be a potential mechanism that mediates the lifespan extension seen in these worms. This is confirmed by our experiments showing that mutation or RNAi of *aakg-4* shortened the lifespan of *daf-2* mutants. Taken together, this implies that AMPK complexes containing the AAKG-4 γ subunit are functionally important downstream of IIS. One possibility is that when IIS is reduced, AAKG-4 replaces other AMPK γ isoforms thereby increasing its capacity to inhibit aging.

Microarray analysis suggests that expression of *aak-2* and *aakb-1* is also promoted by DAF-16, i.e. that there is activation of α, β and γ AMPK subunits. Further analysis of regulation of these 2 genes, e.g. using qRT-PCR and GFP reporter analysis, confirmed this for *aakb-1* (Figure S7B and Figure S9C). However, such activation appears to contribute little to *daf-2* Age (Figure S10). By contrast, *aak-2* is either not regulated by DAF-16, or subject to regulation that is relatively weak and at the limits of detectability and/or condition dependent. This latter result contrasts with the absolute requirement of *aak-2* for *daf-2* longevity and suggests that although this catalytic subunit is essential for AMPK activity [25], [26], IIS regulates AMPK via the γ subunits.

In summary, the α subunit AAK-2 is important for *daf-2* Age [25]–[27] but *aak-2* is not robustly up-regulated by DAF-16; the β subunit AAKB-1 is not important for *daf-2* Age, but is up-regulated by DAF-16; and the γ subunit AAKG-4 is both important for *daf-2* Age and up-regulated directly by DAF-16.

### Atypical AMPK γ isoforms lack AMP binding sequences

Given the high degree of protein sequence conservation, it seems highly likely that worm AMPK, like mammalian AMPK, is regulated by AMP/ADP. However, the observation that *C. elegans* AAKG-4 and AAKG-5 (Figure 1B, Table S2) lack key residues involved in interacting with AMP/ADP in mammals [37] suggests the possibility that AMPK trimers formed with AAKG-4 or AAKG-5 are active in the absence of AMP. Moreover, like AAKG-4 and AAKG-5, budding yeast Snf4 lacks some of the key residues that are important in binding AMP in the human γ1 isoform (e.g. human γ1 R70 and H150) (Figure 1B), and the yeast enzyme is not regulated by AMP. Consistent with the hypothesis that Chromadorean AMPK is AMP independent, we observed that a stress known to increase AMP∶ATP ratio [25] could increase levels of active AMPK in *aakg-4/-5*(RNAi) worms, predicted to contain only AMP-dependent AMPK, but not in *aakg-1/2*(RNAi) worms, predicted to contain only Chromadorean AMPK (Figure 1C). It will be interesting to examine further the enzymology of Chromadorean AMPK, e.g. using *in vitro* analysis of recombinant *C. elegans* proteins.

One further influence of mutating the key nucleotide-interacting residues is that they also cause an increase in the phosphorylation and basal activity of AMPK [39]–[41], [55]. This means that these mutant complexes are effectively constitutively active. Thus, *C. elegans* AMPK complexes containing the AAKG-4 or AAKG-5 γ isoforms may not only be unable to respond to changes in the energy status of the cell but they may also have a higher basal activity compared to AAKG-1, AAKG -2 or AAKG-3-containing AMPK, i.e. show constitutive activity. Consistent with this, worms treated with *aakg-4/-5*(RNAi) but not *aakg-2/-3*(RNAi) did show slightly reduced pAMPKα levels in unstressed, fully fed worms (Figure 1C). Both isoforms are present in nematodes throughout the large Chromadorea clade, implying an important and non-redundant function for these two atypical AMPK γ isoforms.

Up-regulation of AAKG-4 in *daf-2* and the abundant expression of AAKG-5 suggest an interesting speculation: that abundant AMP-independent Chromadorean AMPK in *daf-2* mutants permits a high level of AMPK activity in nutritionally replete animals, where AMP/ATP ratios are low. Possibly this AMPK equivalent of having your cake and eating it contributes to the unusual longevity of *C. elegans* IIS mutants. Consistent with this, *daf-2* Age is partially *aakg-4* dependent. It will be interesting to examine levels of AMPK activity in *daf-2* mutants in future studies.

The presence of an AMP-independent AMPK suggests that fitness among Chromadorean nematodes is enhanced by high levels of AMPK activity under nutritionally replete conditions. In *C. elegans* this might aid entry into the dauer state while food levels are still relatively high. Moreover, AMPK activity might help maintain diapause in developmentally arrested parasitic larvae that exist in a nutritionally replete state, e.g. in microfilariae of filarial worms such as *Onchocerca volvulus*, or second stage larvae of ascarids such as *Toxocara canis*.

### Tissue-specific expression of AMPK subunits

The presence of two α, two β and five γ AMPK subunits in *C. elegans* implies considerable complexity in AMPK function in this organism. Presumably AMPK subunits that are expressed in the same tissues combine to make active trimers. We have used GFP transcriptional reporters to characterize the expression patterns of *aakg-4*, *aakg-5* and *aakb-1* and to verify that of *aak-2* (Table S10). Both *daf-16* and its direct target *aakg-4* show a broad and similar distribution of expression, including several tissues critical for regulating adult lifespan, e.g. the amphid neurons and intestine [56], [57]. In adults the expression of *aak-2* and *aakb-1* appears to be more restricted than that of *aakg-4*, but all 3 are expressed in the intestine (Table S10). If the *C. elegans* γ subunits do have distinct tissue specific roles this would mimic the situation in mammals where e.g. human AMPK γ2 (PRKAG2) is particularly important in cardiac function [58].

It seems likely that there is at least some functional redundancy between subunit isoforms. In dauer larvae *aak-2* can act with either *aakb-1* or *aakb-2* and both β subunits need to be removed to fully abrogate the long lifespan of dauer larvae [59]. Such redundancy may explain why *aakb-1* mutation alone does not affect *daf-2* adult lifespan (Figure S10).

### Reduced IIS generates a DAF-16-AMPK positive feedback loop

AMPK activates DAF-16 [28] and DAF-16 increases expression of several AMPK subunits, suggesting the presence of a positive feedback loop (Figure 5A). Gene regulatory feedback loops have previously been well characterized in unicellular organisms such as *S. pombe* and *E. coli* [60]. Supporting the existence of such a loop, *aakg-4*(RNAi) delays induction of the DAF-16 target gene *sod-3* (Figure 4B). Such a positive feedback loop could, in principle, serve any of several roles: to increase the stability of the activated status of AMPK and DAF-16; to accelerate the induction of DAF-16 target genes; or to increase the final level of expression of DAF-16 target genes after induction. Our findings provide support for the second but not the third scenario, since blunting of DAF-16 target gene induction by *aakg-4*(RNAi) was transient i.e. detected after 24 hr but not 48 hr (Figure 5B). Thus, the presence of the positive feedback loop may facilitate rapid metabolic response to a changing nutrient environment. For example, it might reduce response time for larvae to activate the dauer entry program in a deteriorating environment, thereby increasing fitness.

### Possible evolutionary conservation of FoxO-AMPK interactions

Many aspects of the IIS-regulated signaling network are conserved in higher organisms, and this may extend to AMPK. In *Drosophila*, the AMPK γ subunit SNF4Aγ is up-regulated in flies expressing a dominant negative form of the insulin receptor, although binding of dFOXO to the corresponding promoter is not detected in whole flies [61]. We also explored the possibility that similar relationships exist in mammals but data mining did not reveal any FoxO-dependent changes in AMPK γ subunits in mice [34], [62]–[65]. However, this does not rule out FoxO regulation AMPK gene transcription as these data sets only examine a limited number of tissues. Notably, a recent study showed that human FoxO3 and FoxO4 directly activate LKB1 expression *in vitro* [66]. Given that LKB1 phosphorylation activates AMPK, this suggests that FoxO-AMPK positive feedback loops could be operational in mammals.

### Conclusions

In this study we have carefully mapped out part of the gene regulatory network centered on DAF-16. This has revealed a positive feedback loop involving DAF-16 and AMPK, involving an atypical AMPK γ subunit which may act independently of AMP. Thus, AMPK acts an effector as well as an activator of DAF-16 in its promotion of longevity.

## Materials and Methods

### Strains and strain construction

Worms were maintained at 20°C unless otherwise indicated. The following strains were used: N2, NL2099 *rrf-3(pk1426)*, GA303 *rrf-3(pk1426); daf-2(m577)*, GA1073 *rrf-3(pk1426); daf-16(mgDf50); daf-2(m577)*, GA1061 *aakb-1(tm2658)* (crossed 3 times into recently thawed cultivar of CGC male N2 stock), GA1069 *daf-2(m577); aakb-1(tm2658)*, GA1071 *aakg-4(tm5539)* (crossed 3 times into a recently thawed cultivar of CGC male N2 stock), GA1072 *daf-2(m577); aakg-4(tm5539)*, GA1052 *sEx10615 [Paak-2::gfp]*, GA1048 *daf-2(e1370) sEx10615 [Paak-2::gfp]*, GA1050 *daf-16(mgDf50); daf-2(e1370) sEx10615 [Paak-2::gfp]*, GA1051 *sEx11830 [Paakb-1::gfp]*, GA1047 *daf-2(e1370) sEx11830 [Paakb-1::gfp]*, GA1049 *daf-16(mgDf50); daf-2(e1370) sEx11830 [Paakb-1::gfp]*, GA1403 *wuEx256 [Paakg-4::gfp]*, GA1404 *daf-2(e1370) wuEx256 [Paakg-4::gfp]*, GA1405 *daf-16(mgDf50); daf-2(e1370) wuEx256 [Paakg-4::gfp]*, GA1410 *wuEx257 [Paakg-4::gfp]*, GA1411 *daf-2(e1370) wuEx257 [Paakg-4::gfp]*, GA1412 *daf-16(mgDf50); daf-2(e1370) wuEx257 [Paakg-4::GFP]*, GA1400 *wuEx251 [Paakg-5::gfp]*, GA1401 *daf-2(e1370) wuEx251 [Paakg-5::gfp]*, GA1402 *daf-16(mgDf50); daf-2(e1370) wuEx251 [Paakg-5::gfp]*, GA1413 *wuEx258 [Paakg-5::gfp]*, GA1414 *daf-2(e1370) wuEx258 [Paakg-5::gfp]*, GA1415 *wuEx258 daf-16(mgDf50); daf-2(e1370) [Paakg-5::gfp]*, GA1431 *wuEx261 [Pdaf-16a(i)::gfp]*, GA1433 *daf-2(e1370) wuEx261 [Pdaf-16a(i)::gfp]*, GA1435 *daf-16(mgDf50); daf-2(e1370) wuEx261 [Pdaf-16a(i)::gfp]*, GA1419 *wuEx260 [Pdaf-16a(i)::gfp]*, GA1420 *daf-2(e1370) wuEx260 [Pdaf-16a(i)::gfp]*, GA1421 *daf-16(mgDf50); daf-2(e1370) wuEx260 [Pdaf-16a(i)::gfp]*, GA1437 *wuEx263 [Pdaf-16a(ii)::gfp]*, GA1440 *wuEx263 [Pdaf-16a(ii)::gfp]*, GA1442 *daf-2(e1370) wuEx263 [Pdaf-16a(ii)::gfp]*, GA1438 *daf-16(mgDf50); daf-2(e1370) wuEx264 [Pdaf-16a(ii)::gfp]*, GA1441 *daf-2(e1370) wuEx264 [Pdaf-16a(ii)::gfp]*, GA1443 *daf-16(mgDf50); daf-2(e1370) wuEx264 [Pdaf-16a(ii)::gfp]*.

Multiple mutants were created using standard methodologies and the presence of genomic deletions was tested either by screening for characteristic phenotypes or via PCR. In the case of PCR, genotyping, of *aakb-1(tm2658)*, was carried out by lysis of parent animals using proteinase K (Sigma) and subsequent PCR using the following primers. F:gcaatgtgattaaaagttatggg, In:cgatgataatcagcaaaagacg, R:cagggtatactacacatgtacc. Primers for *aakg-4(tm5539)* were F: agtctctgacacgccgagtt, In: gcacaggcttttagacttcg, R: cgaagtctaaaagcctgtgc.

The *promoter::gfp* transgenes of *daf-16a* were created using Gateway cloning (Invitrogen). The primer sequences that define the region used for *Pdaf-16a(i)* were: F:cctccatcaacaagagcgttc and R:gcaaatgcaacacggagaaaacg; and for the *Pdaf-16a(ii*): F:catgtctcgtgtgcctctcctttcca and R:taacgtcttcgggaatttcagccaaag. The sequences used to define the *daf-16* 3′UTR were F:attctcttcattttgtttcccctggtgttgttcg and R:catcatcatacagtcgcaaatatatttggggg. The *promoter::gfp* fusions for *aakg-4* and *aakg-5* were made as previously described [67] using the PCR to fuse the promoter of interest to GFP and the *unc-54* 3′UTR. The sequences that define the promoter used for *Paakg-4* were F:aaaagacacactcaatttccataaatatat and R:tgataaatgatgatattttgaggttgtgaaa and for *Paakg-5* F:agatcaaaggcttattgtgcatttctattt and R:tctggaaaataaaagcattaaagtgaaaaat. In each case the transgenic strains were created by microinjection of the plasmid (*Pdaf-16*) or PCR product (*Paakg-4* and *5*).

*aakg-1*, *aakg-2* and *aakg-4* RNAi clones were obtained from the Ahringer library and those for *aakg-3* and *aakg-5* were constructed *de novo*. The primer sequences defining the regions used for *aakg-3* were: F:attaaatgcggccgctaaggataggagttcgtaagtatcaattag and R:attaaagacgtcgtaatatcctacgatgtgtcaatatgtacg; and for *aakg-5* F:attaaagtcgacacacaccagcatccgaacaacgtcgtaact and R:attaaagagctcctaaagattcttcagcttgctgcagacatt. The primers contain *Not*I, *Pst*I, *Sal*I and *Sac*I restriction sites, respectively, allowing the resulting PCR fragments to be cloned into pL4440.

### Alteration of *C. elegans* AMP levels and western blot analysis

Adult worms were allowed to lay eggs for 12 hr on plates seeded with *E. coli* HT115 transformed with RNAi plasmids. The resulting progeny were themselves allowed to develop to young adults at 20°C before harvesting in M9 buffer. Worms were then placed either on control plates or plates containing 1 mM sodium azide for 2 hr at 20°C [25]. Sodium azide plates were made by pipetting a stock solution directly onto NGM plates seeded with *E. coli* OP50 72 hr before use.

Following treatment worms were washed off plates using M9, collected by settling and resuspended in 100 µl Phosphosafe extraction reagent (Millipore) supplemented with a protease inhibitor cocktail (Roche). Protein concentration was determined using BCA (Pierce) and 40 µg protein was separated by SDS-gel electrophoresis. Proteins were subsequently transferred to nitrocellulose membrane and probed using a 1∶1000 dilution of phospho-AMPKα (Thr172) antibody (Cell Signaling). β-actin antibody (Santa Cruz) was used as a loading control. Imaging and quantification of bands was carried out using the ImageQuant LAS4000 imaging system and software (GE Healthcare).

### Microscopy

Worms were raised at 15°C, picked at L4 stage, and shifted to 25°C for 24 hr to increase transgenic extrachromosomal array expression and to induce the *daf-2* phenotype in the *daf-2(m577)* 1 day old adults. For each slide, 30–40 worms were mounted in M9+0.2% levamisole on a 2% agarose pad and imaged within the following 30 min. Quantification of GFP expression from transgenic strains was carried out using a Leica DMRXA2 microscope using a GFP filter cube (excitation: 470/40 nm; emission: 525/50 nm), an Orca C10600 digital camera (Hamamatsu) and Volocity image analysis software (Improvision). Low level intestinal autofluorescence was detected in the GFP range in all our strains. For the highly expressed *Paakg-4::gfp* and *Paakg-5::gfp* reporters this did not interfere with our analysis but for *Paak-2::gfp* and *Paakb-1::gfp* which required longer exposure times we corrected for autofluorescence using GFP measurements from non-transgenic animals or by measuring GFP levels only in the head where autofluorescence is minimal.

Confocal images were acquired using the Zen2009 software driving a LSM-710 confocal station with an inverted Axio Observer microscope (Carl Zeiss Microscopy, Germany). Whole worm images were generated from the Z-projection of 12–18 XY-planes acquired every 3 µm through a 10× Plan-Apochromat 0.45 NA dry objective (Carl Zeiss Microscopy, Germany). High-resolution images were generated from the Z-projection of 45–60 XY-planes acquired every 0.75 µm through a 40× Plan-Apochromat 1.3 NA oil objective (Carl Zeiss Microscopy, Germany). Z-projections were created using the freeware LSM5 image browser (Carl Zeiss Microscopy, Germany). Tiff images were then exported to Photoshop CS4 (Adobe), background was subtracted and contrast adjusted for optimal display.

### Quantitative RT-PCR

RNA was isolated from adult worms after transfer of the worms to an unseeded NGM plate to remove *E. coli*. 50–100 worms were used for each assay. RNA was extracted using Trizol (Sigma) and cDNA synthesized using SuperScript II reverse transcriptase with oligo dT (Invitrogen). qRT-PCR was carried out using Fast SYBR Green Master Mix (Applied Biosystems) and the 7900 HT Fast PCR system (Applied Biosystems). Normalization of transcript quantity was carried out using the geometric mean of three stably expressed reference genes Y45F10D.4, *pmp*-3, and *cdc-42* in order to control for cDNA input, as previously described [68]. Primer sequences for DAF-16 target genes are as previously described [53], [54]. Primer sequences for ChIP PCR are as follows: M01H9.3 F:gcatgtgaccacgtgaattt and R:aacccctctaacactatcca; *aakg-4* (peak 1) F:tctcacactcccttcccact and R:gccgtcgtcacaaatactga; *aakg-4* (peak 2) F: aaaaagcgagcaaagcaaaa R: ttccacatttgtcgcacttc; and *aakg-5* (peak 1 primers 1) F:aggacggactgttttgttgc and R:gctcctcgttttcaatgctt. The relative levels of *aakg* isoform mRNA were determined using custom made primers designed and synthesized by Primerdesign. These were optimized to amplify each of the 5 subunits at the same rate, thus allowing relative quantification without the need for an internal standard.

### Chromatin immunoprecipitation

The protocol for chromatin immunoprecipitation was adapted from a previous report [69]. *C. elegans* cultures were grown on plates on OP50 at 20°C. The worms were collected, washed 4× in PBS buffer, frozen in liquid nitrogen and pulverized with a mortar and pestle. The resulting powder was then re-suspended in 8 ml PBS containing 1% formaldehyde and incubated for 20 min with gentle mixing at room temperature. Crosslinking was stopped by addition of 400 µl 2.5 M glycine solution and 20 min further incubation at room temperature. After 4 washes in PBS containing EDTA-free protease inhibitor tablets (Complete, Roche), samples were flash frozen and stored at −80°C. After thawing, 2 ml of HLB buffer [50 mM HEPES-KOH, pH 7.5, 150 mM NaCl, 1 mM EDTA, 0.1% (wt/vol) sodium deoxycholate, 1% (vol/vol) Triton X-100, 0.1% (wt/vol) SDS and 1× Complete protease inhibitor] was added and sonication was carried out at 70% intensity for 7 bursts of 30 s in a Vibracell sonicator (Sonics). Protein quantity was estimated by BCA (Pierce) and 2 mg were diluted to 500 µl in HLB buffer.

3×50 µl aliquots were removed at this point. DNA isolated from these samples was subsequently used for input controls. Samples were pre-cleared for 1 hr in prewashed salmon sperm DNA/protein-A agarose beads (Millipore) and then incubated overnight with 10 µl of anti-DAF-16 Ab (Santa Cruz). Samples were then incubated with prewashed salmon sperm DNA/protein-A agarose beads for 2 hr. The beads were then washed twice in WB1 [50 mM HEPES-KOH, pH 7.5, 150 mM NaCl, 1 mM EDTA, 1% (wt/vol) sodium deoxycholate, 1% (vol/vol) Triton X-100, 0.1% (wt/vol) SDS and 1× Complete protease inhibitor], twice in WB2 [50 mM HEPES-KOH, pH 7.5, 1 M NaCl, 1 mM EDTA, 1% (wt/vol) sodium deoxycholate, 1% (vol/vol) Triton X-100, 0.1% (wt/vol) SDS and 1× Complete protease inhibitor] and once in WB3 [50 mM Tris-HCl, pH 8, 0.25 mM LiCl, 1 mM EDTA, 0.5% (vol/vol) NP-40 and 0.5% (wt/vol) sodium deoxycholate]. Crosslinking was reversed by addition of proteinase K solution [50 mM Tris-HCl, pH 8, 25 mM EDTA, 1.25% (wt/vol) SDS, 160 µg/ml proteinase K (Qiagen)] and incubation for 2 hr at 45°C and overnight at 65°C. DNA was isolated by applying solution to Qiagen PCR purification columns.

### Lifespan analysis

Prior to experiments animals were maintained at the permissive temperature and grown for at least one generation in the presence of food to assure full viability. Lifespan assays were performed essentially as described [70]. Survival plots and *p* values (log rank test) were determined using JMP software, version 7. Cox proportional hazard *p* values were determined in R from combined experiments using mixed effects models (Christensen, R. H. B., 2012 http://www.cran.r-project.org/package=ordinal). For lifespans using RNAi worms were grown on bacteria expressing *aakg* RNAi clones from the L4 stage. Empty pL4440 vector was used as a control.

### Bioinformatics

For the majority of species orthologs of the *C. elegans* genes *aakg-1-5* were identified by local alignment searches of the five protein sequences to the gene models in WormBase. Since not all genomes were available in WormBase orthologs for the parasitic helminths (*B. xylophilus*, *E. granulosus*, *E. multilocularis*, *T. solium*, *H. microstoma*, *S. mansoni* and *S. japonicum*) were derived from GeneDB. Orthologs for *G. pallida*, *H. contortus*, *O. volvulus*, *S. ratti* and *T. muris* were derived using data available on the Sanger Institute Scientific Resources website. Since there are no gene model predictions available for *O. volvulus* and *T. muris*, we identified contigs that aligned closely to the *C. elegans* gene sequences of interest using a “protein versus translated DNA” Blast search. We then predicted genes within these contigs using the Eukaryotic GeneMark.hmm [71]. Of the several genes predicted, we kept for further analysis the ones with protein sequences that showed high similarity to the *C. elegans aakg* genes. Finally, orthologs of *P. pacificus* were derived from the gene predictions available from www.pristionchus.org.

Multiple sequence alignments of the protein sequences were done using MUSCLE [72]. Trees were constructed with ClustalW2 Phylogeny [73] using the Neighbour-joining method. The species tree was constructed by integrating the taxonomy information available through WormBase, Uniprot Taxonomy [74] and PhyloExplorer [75] (based on data from TreeBASE). All trees were visualized and annotated using EvolView [76].

## Supporting Information

Figure S1 Relationship between the AMPK γ isoforms in the Nematoda and Platyhelminthes. This phylodendrogram supports the model that the AAKG-4 and AAKG-5 isoforms diverged from a single ancestral gene in an early ancestor of the Chromadorea, i.e. the common ancestor of *C. elegans* and *B. malayi*. It is possible that the presence of atypical AAKG isoforms in the Enoplid nematodes *T. muris* and *T. spiralis* reflects the existence of a primordial atypical isoform in the common ancestor of the Chromadorea and Enoplea. The earlier existence of this atypical isoform is unclear: Parasitic platyhelminthes contain a divergent AAKG isoform in which the AMP-binding residues are largely conserved (Table S3). One platyhelminth, *S. mansoni*, possesses an atypical isoform (144700.1) that clusters with the AAKG-4/5 group, but this is exceptional (Table S3).
(PDF)

Figure S2 Relationship between γ isoforms in the Rhabditida. Representatives of the 5 AAKG isoforms are identifiable in most Rhabditid species. An exception is *C. japonica*, which lacks an *aakg-5* ortholog.
(PDF)

Figure S3 Evolutionary relationship between nematode and platyhelminth lineages. Species shown are represented in Figure 1A, S1 and S2.
(PDF)

Figure S4 Immuno-precipitation and Western blot with anti-DAF-16 antibody. Immuno-precipitation was carried out as for ChIP using the anti-DAF-16 ce-300 antibody (rabbit polyclonal from Santa Cruz). The resulting lysates were separated on an SDS gel and transferred to nitrocellulose membrane before probing with a second DAF-16 antibody raised in a different species to avoid cross-reactivity (anti-DAF-16 c-N goat polyclonal from Santa Cruz).
(PDF)

Figure S5 DAF-16 binding profiles in four AMPK genes. Peaks represent sites of increased DNA methylation caused by binding of DAF-16::Dam methylase fusion protein (DamID). Plots generated using data from a previous study [22], in which we did not detect DAF-16 binding to the promoter of *aak-2* or *aakb-1*. In that study to avoid false positives we applied strict criteria to define DAF-16 binding sites, including location no more than 1 Kb from the predicted translational start site. However, a potential DAF-16 binding site is present in the *aakb-1* promoter 1.5 Kb from the start site, in the vicinity of a weak DAF-16 binding element (DBE) [48] suggesting that this gene is directly regulated by DAF-16. DAF-16 binding peaks or DBEs were not detected in the *aak-2* promoter.
(PDF)

Figure S6 *aakg-5* is broadly expressed but its transcription is not regulated by IIS. A) Confocal images showing the *Paakg-5::GFP* expression pattern in 1-day old hermaphrodites. The *Paakg-5::GFP* transgene was created using the fusion method of PCR to ‘stitch’ together 1.85 Kb of promoter taken directly upstream of the transcriptional start site, GFP and the *unc-54* 3′UTR [79]. This PCR product was then introduced as an extra-chromosomal array into N2 worms and two independent transgenic strains were isolated for each gene. (i) Whole worm expression pattern. (ii) *Paakg-5::GFP* is expressed in the female gonad sheath cells (GonSh), vulva epithelium (VlvE) and neurons (VlvN), ventral cord neurons (VC) and excretory cell (Exc). (iii) It is also seen in the spermatheca (Sperm) and epithelial seam cells (Seam). (iv) In addition to the excretory cell (Exc), *Paakg-5::GFP* displays strong expression levels in the pharyngeal epithelia (PhxE), neurons (PhxN), some ring neurons (RingN) and sensory neuron (SensN) termini. (v) In the tail, *Paakg-5::GFP* signal mostly localizes to the pre-anal ganglion (RectN), rectum epithelium (RectE), intestinal-rectal valve (RectV) and phasmid support cells (PhaSh, PhaSc). As seen along the whole worm, it is also clearly expressed in the seam cells (Seam), intestine (Int) and excretory cell arms (Exc). Table S10 compares expression of *Paakg-5::gfp* with other AMPK subunits. B) Quantification of GFP fluorescence in worms expressing a *Paakg-5::GFP* reporter. We did not observe any differences in GFP fluorescence levels when our transgene was crossed into *daf-2* or *daf-16; daf-2* backgrounds. The expression pattern was also unchanged (data not shown). The same was also true for a second set of strains generated from a different extrachromosomal array. Error bars, standard deviation. C) qRT-PCR of *gfp* mRNA in worms expressing a *Paakg-5::GFP* reporter did not reveal any change in expression between *daf-2* and *daf-2; daf-16*. Error bars, standard deviation.
(PDF)

Figure S7 Evidence of regulation of *aakb-1* by insulin/IGF-1 signaling. qRT-PCR data. A, B) *aakb-1* but not *aak-2* mRNA levels are increased in *daf-2* animals in a *daf-16*-dependent manner. ^♦^p<0.08 compared to N2, *p<0.08 compared to *daf-2*. Error bars, standard deviation.
(PDF)

Figure S8 Evidence of regulation of *aak-2* by insulin/IGF-1 signaling. A) Confocal images showing *Paak-2::GFP* expression pattern in 1-day old wild type hermaphrodites. The *Paak-2::gfp* sequence in *sEx10615* contains 2.821 Kb of DNA upstream of the transcriptional start site. (i) Whole worm expression pattern. (ii) Head *Paak-2::GFP* expression is mostly seen in the excretory cell (Exc) but is also observed in pharyngeal neurons (PhxN), epithelial cells (PhxE), a subset of ring neurons (RingN), amphid socket cells (AmphSc) and head body wall muscles (HM). (iii) *Paak-2::GFP* is also expressed in the posterior intestine (Int), rectal gland (RectG) and epithelial cells (RectE), and in phasmids (Pha). (iv) *Paak-2::GFP* is detected in vulval muscles (VlvM). This expression pattern builds on but is consistent with previous observations of this strain [51] and another previous study [80] which used a *Paak-2::aak-2::gfp* translational reporter. Table S10 compares expression of *Paak-2::gfp* with other AMPK subunits. B) Quantification of GFP fluorescence in worms expressing a *Paak-2::GFP* reporter. We did not observe any differences in GFP fluorescence levels when our transgene was crossed into *daf-2* or *daf-16; daf-2* backgrounds. The expression pattern was also unchanged (data not shown). GFP fluorescence values are corrected for background intestinal autofluorescence using values obtained from non-transgenic animals (see methods). Error bars, standard deviation. C) qRT-PCR of *gfp* mRNA in worms expressing a *Paak-2::GFP* reporter revealed a small, significant change in expression between *daf-2* and *daf-2; daf-16*. ^♦^p<0.05 compared to N2, *p<0.05 compared to *daf-2*. Error bars, standard deviation.
(PDF)

Figure S9 Expression pattern of *aakb-1*. A) Confocal images showing *Paakb-1::GFP* in 1-day old wild type hermaphrodites. The *Paakb-1::gfp* reporter contains 2.845 Kb of promoter directly upstream of the transcriptional start site. (i) Whole worm expression pattern. (ii) *Paakb-1::GFP* is strongly expressed in pharyngeal muscles (PhxM), the pharyngeal intestinal valve cells (VPI) as previously noted [51] and a few head neurons of the pharynx (PhxN) and the ring region (RingN). It is also detected in some support cells (SC). (iii) Tail expression of *Paakb-1::GFP* includes the tail minor epithelium (TE), the rectal-intestinal valve (RectV), posterior intestine and body wall muscles (BWM). (iv) Mid-body expression is mostly restricted to muscles of the vulva (VlvM), uterus (UtM) and body wall (BMW), but is also detected in the intestine (Int). However we noted that the levels of GFP expression in all genetic backgrounds was highly variable between animals. Table S10 compares expression of *Paakb-1::gfp* with other AMPK subunits. B) Quantification of GFP fluorescence in the heads of worms expressing a *Paakb-1::GFP* reporter. The *daf-16; daf-2* background carrying this transgene exhibited unexplained and higher than normal intestinal autofluorescence making fluorescence quantification of whole worms difficult. To circumvent this we measured GFP fluorescence levels in the heads of worms, where *Paakb-1::GFP* is highly expressed. Mean of four trials shown. Error bars, standard deviation. C) qRT-PCR of *gfp* mRNA in worms expressing a *Paakb-1::GFP* reporter revealed an increase in expression between *daf-2* and *daf-2; daf-16*, however this was not significant, probably due to transgene expression varying between individuals. Error bars, standard deviation.
(PDF)

Figure S10 *aakb-1* does not contribute to *daf-2* longevity. The proportional reduction in lifespan caused by *aakb-1(tm2658)* does not significantly differ between *daf-2(+)* and *daf-2(m577)* backgrounds. The combined data from 4 trials is shown (C in Table S6).
(PDF)

Figure S11 *aakg-4(tm5539)* does not alter progeny production. The total progeny were counted from individual adult worms. This graph represents data from two independent trials. n = 20 broods for each genotype, * *p*<0.05 compared to N2.
(PDF)

Figure S12 *aakg-5* RNAi shortens *daf-2* lifespan. One representative experiment is shown corresponding to trial 9 in Table S8. Reduction of *aakg-5* mRNA was confirmed by qRT-PCR (data not shown). However, *aakg-5* RNAi did not further reduce the lifespan of *daf-2(m577);aakg-4(tm5539)* double mutants.
(PDF)

Figure S13 Induction of the DAF-16 target gene *sod-3* in *daf-2* mutants. A) L4 *daf-2(m577)* animals were shifted to 25°C prior to RNA extraction. B) *sod-3* mRNA levels are induced in response to temperature specifically in *daf-2* mutants.
(PDF)

Figure S14 Effect of *aakg* RNAi on induction of *sod-3* expression by reduced IIS. A) Experimental protocol. L4 *daf-2(m577)* animals were treated with *aakg* RNAi and shifted to 25°C prior to RNA extraction (Figure 4A). B) *sod-3* mRNA levels are induced in response to temperature but this induction is not reduced by *aakg-1*, *aakg-2* or *aakg-5* RNAi. The level of *sod-3* at 0 hr is that of adults that have not been shifted to 25°C i.e. maintained at 15°C. Error bars, standard deviation. These RNAi treatments were carried out in parallel with *aakg-4* RNAi (Figure 4B). C) Statistical analysis of data generated on pooled data sets for each time point and RNAi treatment.
(PDF)

Figure S15 Effects of *aakg* RNAi on DAF-16::GFP nuclear localization. We created a strain that expressed the DAF-16a::GFP transgene [81] in a *daf-2(e1370)* background. *daf-2(e1370)* is a particularly strong class 2 *daf-2* allele, and DAF-16::GFP shows exclusively nuclear localization at 25°C. However, at A) 15°C or B) 20°C (temperatures where this allele still extends lifespan), weaker DAF-16::GFP nuclear localization is seen [82]. 70–100 animals (per group) were scored for DAF-16::GFP nuclear localization using a scoring system similar to that used in [83]. *p*, Chi Squared test *vs* control.
(PDF)

Figure S16 DAF-16 does not regulate its own transcription. A) qRT-PCR shows that *daf-16* mRNA levels are affected in a *daf-2* background by *daf-16(mgDf50)* (a deletion allele) but not by *daf-16(m26)* (a point mutant allele). **p<0.002, *p = 0.03. B) It remains possible that *daf-16* activates its own expression in some worm tissues or cell types, but that this is not detectable in whole worm mRNA extracts. To test this we created and examined *Pdaf-16a::gfp* transcriptional reporters. Given the large size of the predicted DAF-16-binding region, 2 types of *Pdaf-16a::gfp* reporters, *i* and *ii*, were created containing overlapping 5′ and 3′ regions of the promoter *daf-16a::GFP* transgenes were designed based on DAF-16 binding sites predicted by DamID and modENCODE. Transgenes were introduced as extrachromosomal arrays into wild-type worms and two independent transgenic lines for each construct were isolated. In a wild-type background, both constructs led to near ubiquitous GFP expression patterns similar to those previously reported for DAF-16a::GFP translational reporters [6], [53], [81]. C, D) Quantification of GFP fluorescence in worms expressing *Pdaf-16a(i)::GFP* or *Pdaf-16a(ii)::GFP* reporters. We observed a decrease in GFP fluorescence levels when our transgene was crossed into *daf-2* which was reversed by *daf-16*. The same was also true for a second set of strains generated from different extrachromosomal arrays. The expression pattern was unchanged (data not shown). ^♦^ *p*<0.01 compared to N2 *p<0.01 compared to *daf-2*. Error bars, standard deviation. E, F) Quantification of *gfp* mRNA in worms expressing *Pdaf-16a(i)::GFP* or *Pdaf-16a(ii)::GFP* reporters. ^♦^ *p*<0.01 compared to N2, *p<0.01 compared to *daf-2*. Error bars, standard deviation. In worms with *Pdaf-16a(i)::gfp* reporters, GFP fluorescence levels were slightly decreased in *daf-2(e1370)* compared to *daf-16(mgDf50); daf-2* backgrounds, but *gfp* mRNA levels were not (Figure S16C and S16E). However, in worms with *Pdaf-16a(ii)::gfp* reporters, GFP fluorescence and *gfp* mRNA were both reduced in *daf-2* animals compared to *daf-16; daf-2* (Figure S16D and S16F). These results confirm that *daf-16* does not activate its own expression, and even suggest some degree of auto-inhibition of expression.
(PDF)

Table S1 Percentage amino acid sequence similarity of *C. elegans* AMPK γ subunit CBS domains to human PRKAG1.
(PDF)

Table S2 AMP/ATP binding residues in Rhabditida. All sequences obtained were aligned using ClustalW with an identity matrix. Residues known to be involved in nucleotide binding were identified using the human PRKAG1 gene. Atypical residues are in red. Grey blocks show sequences that cluster with *C. elegans* atypical isoforms *aakg-4* and *aakg-5* (Figure S2).
(PDF)

Table S3 AMP/ATP binding residues in Nematoda and Platyhelminthes. All sequences obtained were aligned simultaneously using ClustalW with an identity matrix. Residues known to be involved in nucleotide binding were identified using the human PRKAG1 gene. Atypical residues are in red. Grey blocks show isoforms that cluster with *C. elegans* atypical *aakg-4* and *aakg-5* which all contain atypical residues. A number of the γ subunit isoforms from various more distant species contain missing or truncated cystathione-β-synthase (CBS) domains, it is possible that these may be pseudogenes. Alternatively, in AMPK complexes containing these isoforms, enzyme function may be compromised by lacking these CBS domains. However, it is possible that only two CBS domains can come together to form the nucleotide-binding region, as seen in other CBS-domain containing proteins [84]. It remains to be seen whether in this situation the absence of conserved nucleotide-interacting residues would allow the enzyme to respond to changes in the AMP/ATP ratio.
(PDF)

Table S4 Effects of insulin/IGF-1 signaling on AMPK gene expression. Fold changes in mRNA levels are shown. Some *p* values are adjusted and some are not, those in bold are considered significant by the authors. For studies comparing *daf-2* with *daf-16(mgDf50);daf-2* values are not noted for *daf-16* as probe sets fall within the boundaries of the *mgDf50* deletion. McElwee *et al.*, 2003: the numbers are an average of four replicates and SD = standard deviation. Budovskaya *et al.*, 2008: negative numbers indicate that the gene is positively regulated by DAF-16. Values represent a total difference across Stanford and Caltech arrays. No differences in *aak-1, aakb-1* or *aakg-1-3* mRNA levels were detected by any of these studies.
(PDF)

Table S5 Statistical analysis of ChIP data. The % input for three individual trials was compared for the two *aakg-4* peaks and one *aakg-5* peak (Figure S5). *aakg-4* peak 2 and *aakg-5* peak 1 were in the promoter fragments that we examined in more detail. Only the *aakg-4* peak contains DAF-16 binding elements. Each experiment was analysed individually and then the data sets combined and tested for significant differences using a mixed effects linear model (MELM). The values obtained for a positive control DAF-16 target gene *M01H9.3* [22] are also shown.
(PDF)

Table S6 Effects of *aakb-1(tm2658)* on lifespan. *aakb-1(tm2658)* shortens lifespan modestly and to a similar degree in *daf-2(+)* and *daf-2(−)* backgrounds. This argues against a role for increased *aakb-1* expression as a cause of *daf-2* Age. C, data from all four trials combined. N, number of worms (total worms scored including those censored). Lifespan assays all carried out at 25°C. *p*, Probability (determined by log rank test) of being the same as ^a^ N2 or ^b^ *daf-2(m577)*. % difference in mean lifespan compared to ^a^ N2 or ^b^ *daf-2(m577)*. SEM, standard error of the mean.
(PDF)

Table S7 *aakg-4(tm5539)* shortens *daf-2* lifespan. N, number of worms (Total worms scored including those censored). *p*, Probability (determined by log rank test) of being the same as ^a^ *daf-2(m577)*, ^b^ N2 or ^c^ *aakg-4(tm5539)*. % difference in mean lifespan compared to *daf-2(m577)*. SEM, standard error of the mean.
(PDF)

Table S8 *aakg-4* and *aakg-5* RNAi shorten *daf-2* lifespan. N, number of worms (total worms scored including those censored). The *rrf-3* mutation sensitizes the animals to the effects of RNAi [52]. Lifespan assays all carried out at 25°C. It was confirmed by qRT-PCR that the appropriate *aakg* mRNA levels were reduced by *aakg-4* or *aakg-5*(RNAi) (data not shown). In the case of *aakg-5* RNAi we sometimes observed an additional decrease in *aakg-4* mRNA levels in worms that had been fed RNAi for longer times suggesting that there may be some cross RNAi that could affect lifespan. *p*, probability (determined by log rank test) of being the same as: ^a^ *rrf-3(pk1426); daf-2(m577)* or *daf-2(m577)* control RNAi. ^b^ *rrf-3(pk1426)* or N2 control RNAi. ^c^ *aakg-4(tm5539)*. ^d^ *daf-2; aakg-4(tm5539)*. % difference in mean lifespan compared to ^a^ *rrf-3(pk1426)* or N2. ^b^ *rrf-3(pk1426); daf-2(m577) or daf-2(m577)*. ^c^ *aakg-4(tm5539)*. ^d^ *daf-2; aakg-4(tm5539)*. SEM = standard error of the mean. NS, not significant (*p*>0.05).
(PDF)

Table S9 RNAi targeted to *aakg-1*, *aakg-2* or *aakg-3* does not consistently suppress *daf-2* mutant longevity. N, number of worms (total worms scored including those censored). Lifespan assays all carried out at 25°C. The *rrf-3* mutation sensitizes the animals to the effects of RNAi [52]. *p*, probability (determined by log rank test) of being the same as ^a^ *rrf-3; daf-2* control RNAi or ^b^ *rrf-3* Control RNAi. % differences in mean lifespan are compared to *daf-2(m577)*. SEM: standard error of the mean.
(PDF)

Table S10 Expression pattern of AMPK subunits. Y = expression observed in this tissue. Dark grey boxes highlight tissues where *Paak-2::GFP*, *Paakb-1::GFP* and *Paakg-4::GFP* expression was observed.
(PDF)
