# Supplementary figures and images for: DAF-16/FoxO Directly Regulates an Atypical AMP-Activated Protein Kinase Gamma Isoform to Mediate the Effects of Insulin/IGF-1 Signaling on Aging in Caenorhabditis elegans
Source: PLoS Genet. 2014 Feb 6;10(2):e1004109. doi: 10.1371/journal.pgen.1004109 (PMC3916255; doi:10.1371/journal.pgen.1004109)

Figure S1

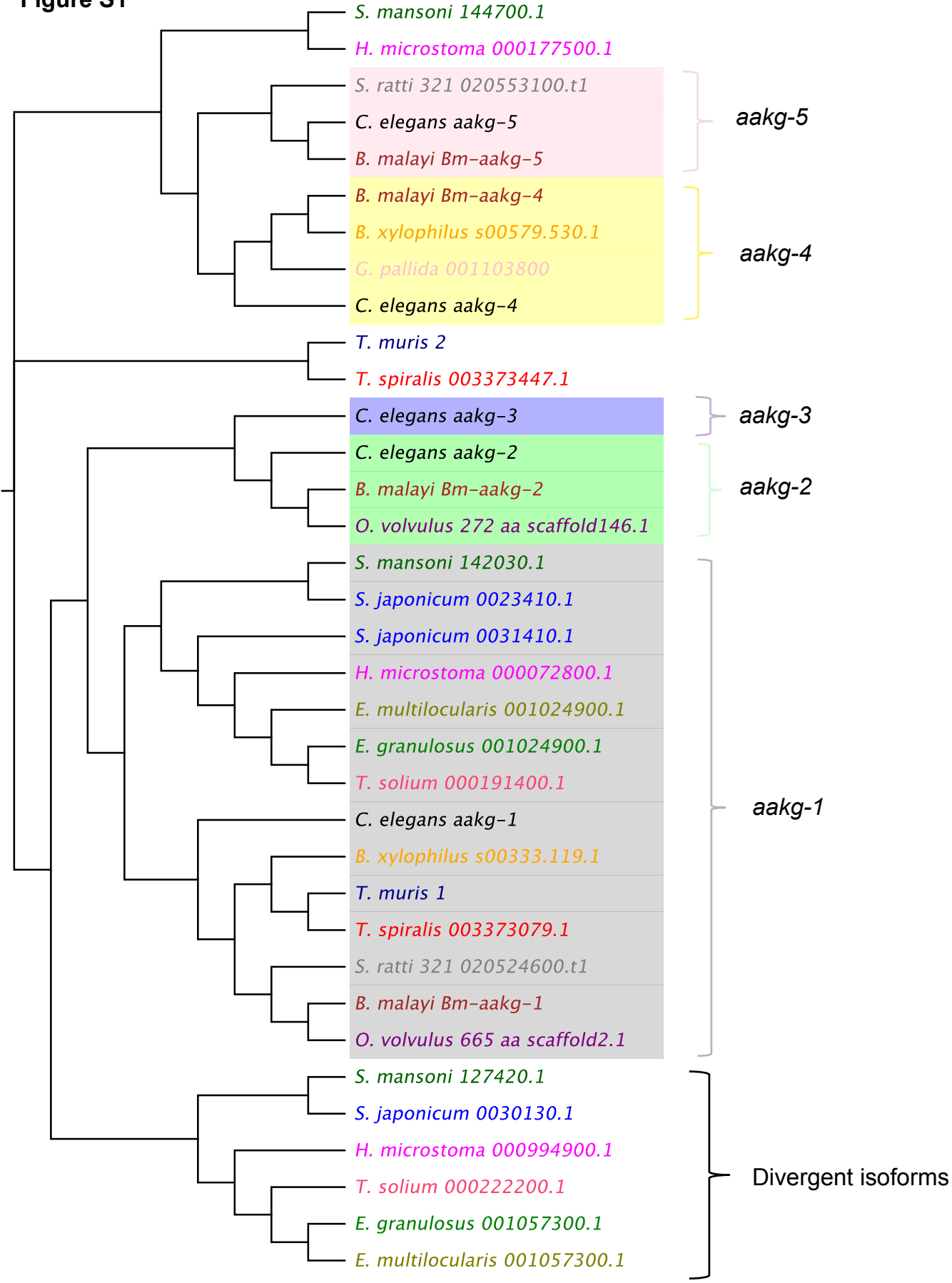

Supplement: Figure S1 — Relationship between the AMPK γ isoforms in the Nematoda and Platyhelminthes. This phylodendrogram supports the model that the AAKG-4 and AAKG-5 isoforms diverged from a single ancestral gene in an early ancestor of the Chromadorea, i.e. the common ancestor of C. elegans and B. malayi. It is possible that the presence of atypical AAKG isoforms in the Enoplid nematodes T. muris and T. spiralis reflects the existence of a primordial atypical isoform in the common ancestor of the Chromadorea and Enoplea. The earlier existence of this atypical isoform is unclear: Parasitic platyhelminthes contain a divergent AAKG isoform in which the AMP-binding residues are largely conserved (Table S3). One platyhelminth, S. mansoni, possesses an atypical isoform (144700.1) that clusters with the AAKG-4/5 group, but this is exceptional (Table S3). (PDF) [file pgen.1004109.s001.pdf]

Figure S2

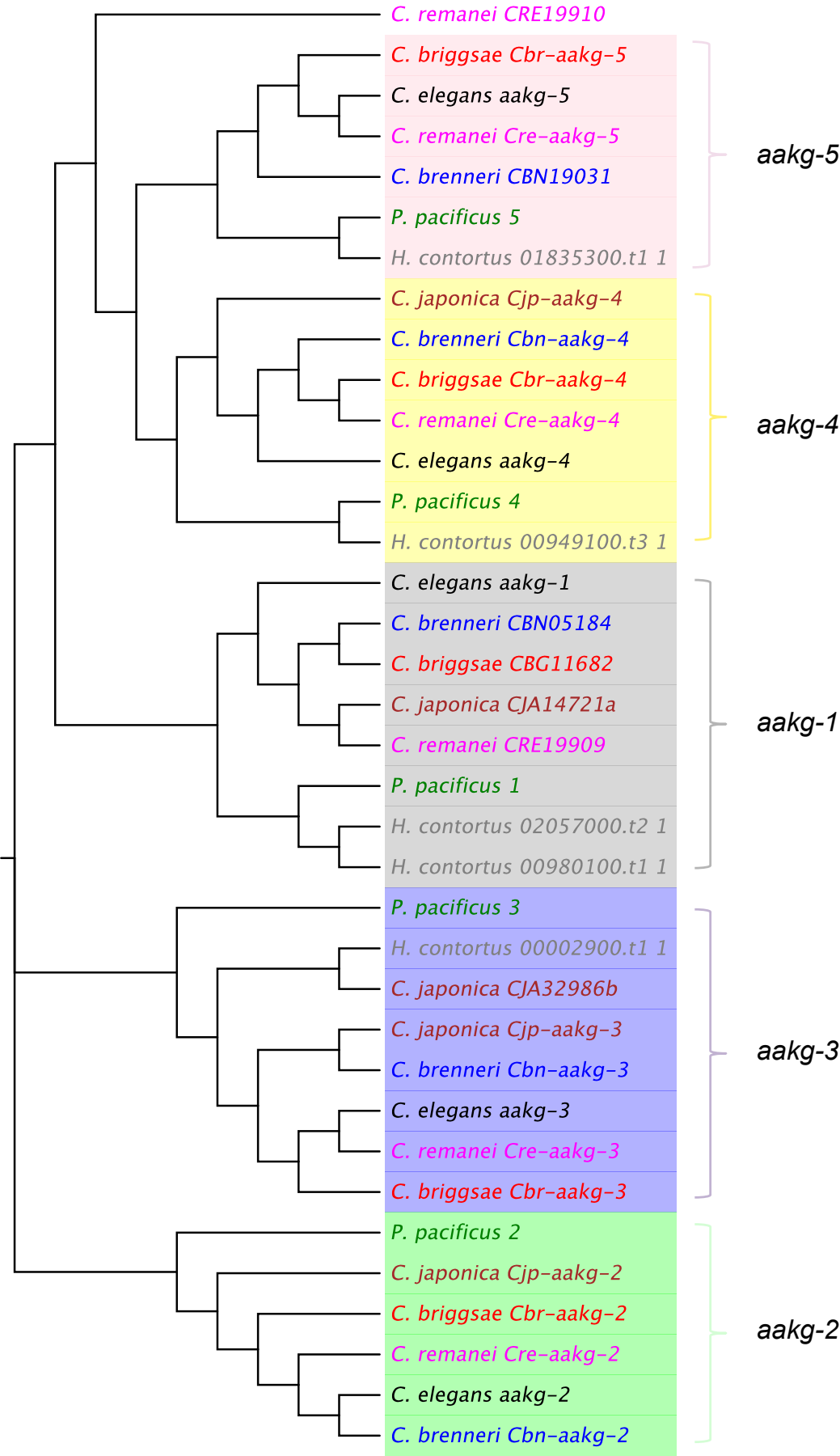

Supplement: Figure S2 — Relationship between γ isoforms in the Rhabditida. Representatives of the 5 AAKG isoforms are identifiable in most Rhabditid species. An exception is C. japonica, which lacks an aakg-5 ortholog. (PDF) [file pgen.1004109.s002.pdf]

**Figure S4**

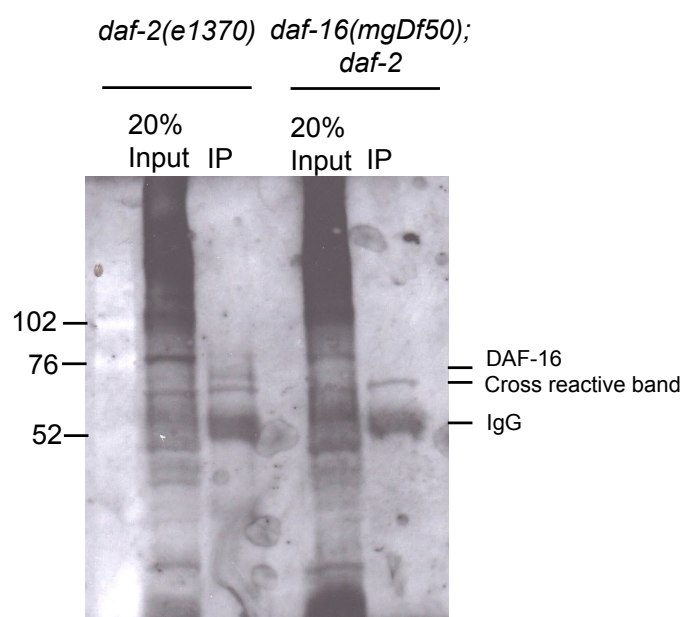

Supplement: Figure S4 — Immuno-precipitation and Western blot with anti-DAF-16 antibody. Immuno-precipitation was carried out as for ChIP using the anti-DAF-16 ce-300 antibody (rabbit polyclonal from Santa Cruz). The resulting lysates were separated on an SDS gel and transferred to nitrocellulose membrane before probing with a second DAF-16 antibody raised in a different species to avoid cross-reactivity (anti-DAF-16 c-N goat polyclonal from Santa Cruz). (PDF) [file pgen.1004109.s004.pdf]

Figure S5

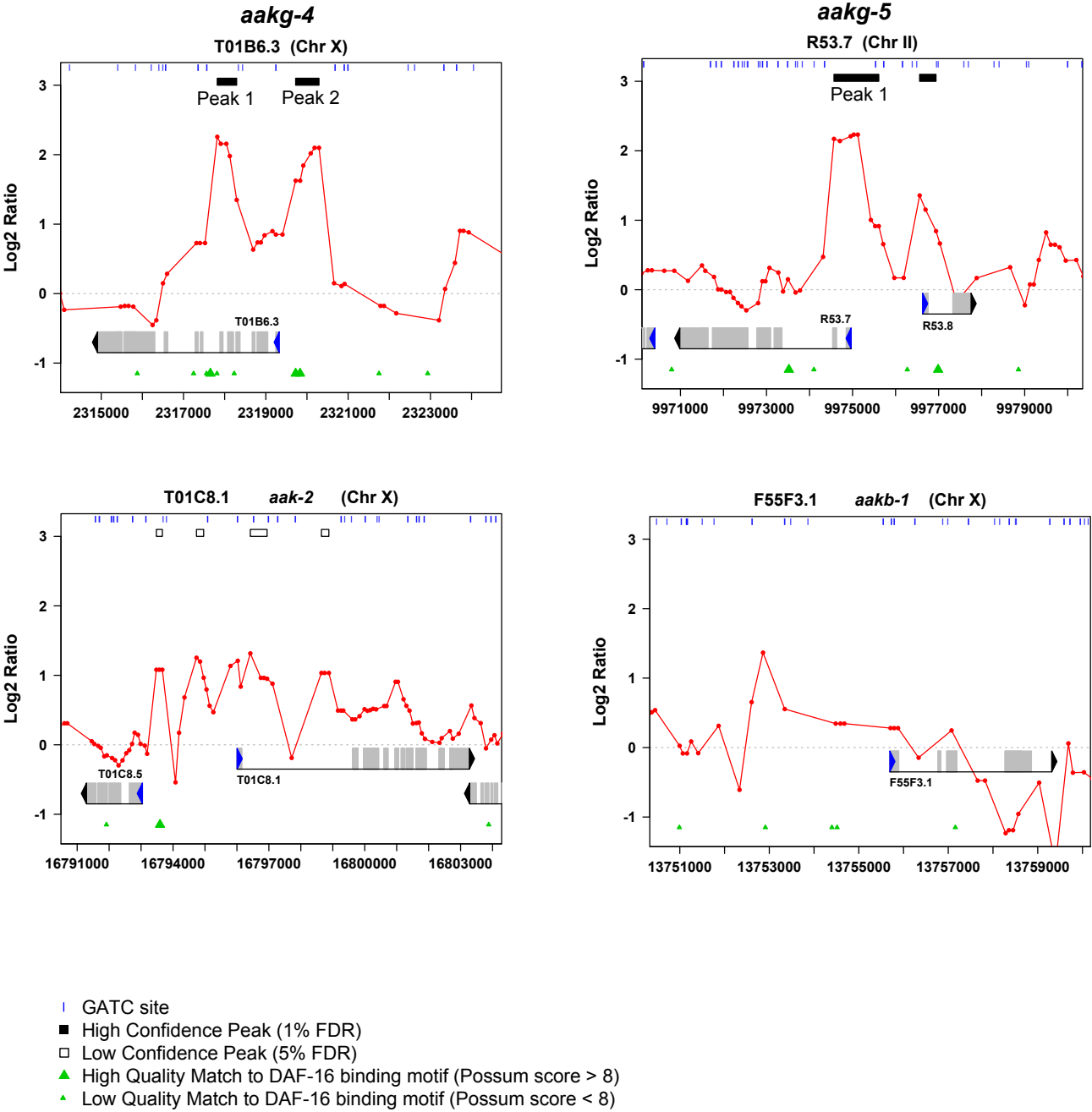

Supplement: Figure S5 — DAF-16 binding profiles in four AMPK genes. Peaks represent sites of increased DNA methylation caused by binding of DAF-16::Dam methylase fusion protein (DamID). Plots generated using data from a previous study [22], in which we did not detect DAF-16 binding to the promoter of aak-2 or aakb-1. In that study to avoid false positives we applied strict criteria to define DAF-16 binding sites, including location no more than 1 Kb from the predicted translational start site. However, a potential DAF-16 binding site is present in the aakb-1 promoter 1.5 Kb from the start site, in the vicinity of a weak DAF-16 binding element (DBE) [48] suggesting that this gene is directly regulated by DAF-16. DAF-16 binding peaks or DBEs were not detected in the aak-2 promoter. (PDF) [file pgen.1004109.s005.pdf]

Figure S6

A

*wuEx251*[*Paakg-5::GFP*]

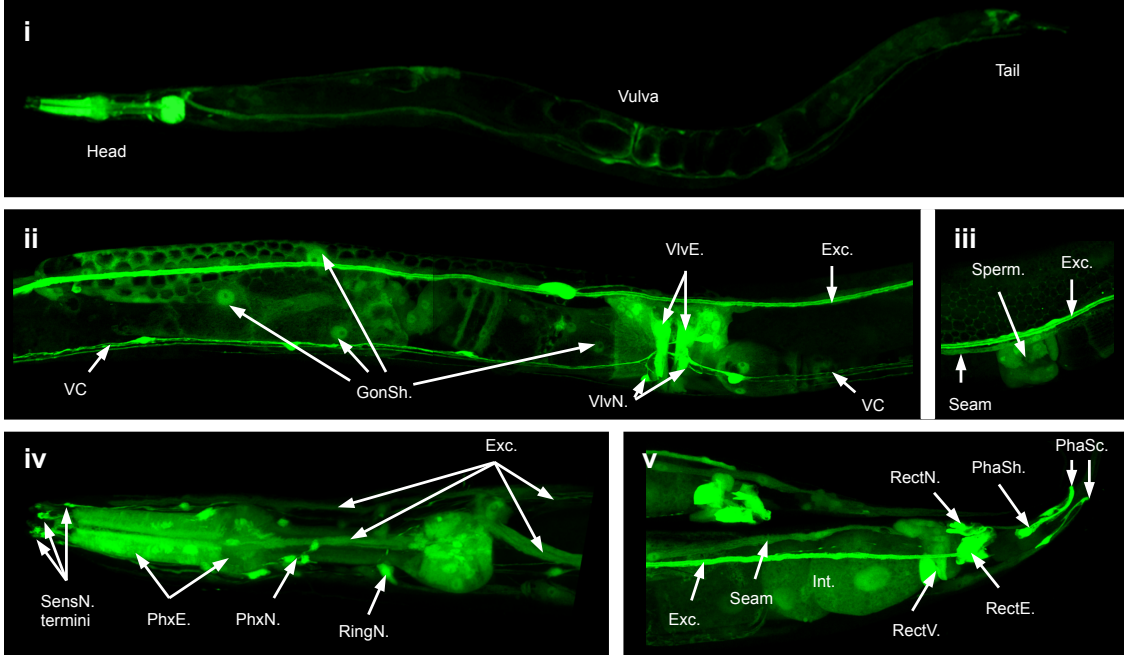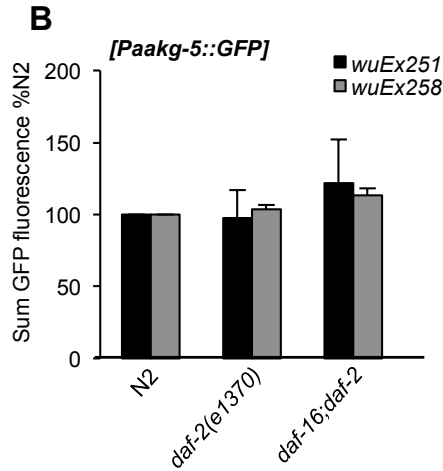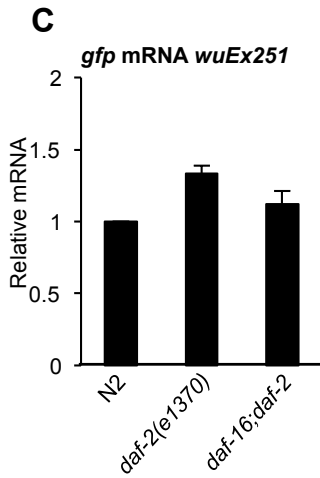

Supplement: Figure S6 — aakg-5 is broadly expressed but its transcription is not regulated by IIS. A) Confocal images showing the Paakg-5::GFP expression pattern in 1-day old hermaphrodites. The Paakg-5::GFP transgene was created using the fusion method of PCR to ‘stitch’ together 1.85 Kb of promoter taken directly upstream of the transcriptional start site, GFP and the unc-54 3′UTR [79]. This PCR product was then introduced as an extra-chromosomal array into N2 worms and two independent transgenic strains were isolated for each gene. (i) Whole worm expression pattern. (ii) Paakg-5::GFP is expressed in the female gonad sheath cells (GonSh), vulva epithelium (VlvE) and neurons (VlvN), ventral cord neurons (VC) and excretory cell (Exc). (iii) It is also seen in the spermatheca (Sperm) and epithelial seam cells (Seam). (iv) In addition to the excretory cell (Exc), Paakg-5::GFP displays strong expression levels in the pharyngeal epithelia (PhxE), neurons (PhxN), some ring neurons (RingN) and sensory neuron (SensN) termini. (v) In the tail, Paakg-5::GFP signal mostly localizes to the pre-anal ganglion (RectN), rectum epithelium (RectE), intestinal-rectal valve (RectV) and phasmid support cells (PhaSh, PhaSc). As seen along the whole worm, it is also clearly expressed in the seam cells (Seam), intestine (Int) and excretory cell arms (Exc). Table S10 compares expression of Paakg-5::gfp with other AMPK subunits. B) Quantification of GFP fluorescence in worms expressing a Paakg-5::GFP reporter. We did not observe any differences in GFP fluorescence levels when our transgene was crossed into daf-2 or daf-16; daf-2 backgrounds. The expression pattern was also unchanged (data not shown). The same was also true for a second set of strains generated from a different extrachromosomal array. Error bars, standard deviation. C) qRT-PCR of gfp mRNA in worms expressing a Paakg-5::GFP reporter did not reveal any change in expression between daf-2 and daf-2; daf-16. Error bars, standard deviation. (PDF) [file pgen.1004109.s006.pdf]

Figure S7

A

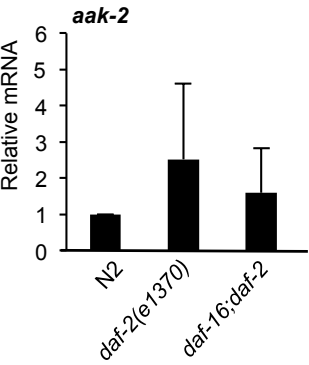

B

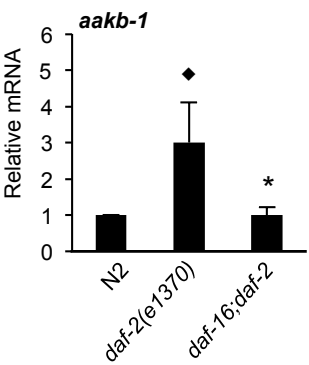

Supplement: Figure S7 — Evidence of regulation of aakb-1 by insulin/IGF-1 signaling. qRT-PCR data. A, B) aakb-1 but not aak-2 mRNA levels are increased in daf-2 animals in a daf-16-dependent manner. ♦p<0.08 compared to N2, *p<0.08 compared to daf-2. Error bars, standard deviation. (PDF) [file pgen.1004109.s007.pdf]

Figure S8

A

*sEx10615*[*Paak-2::GFP*]

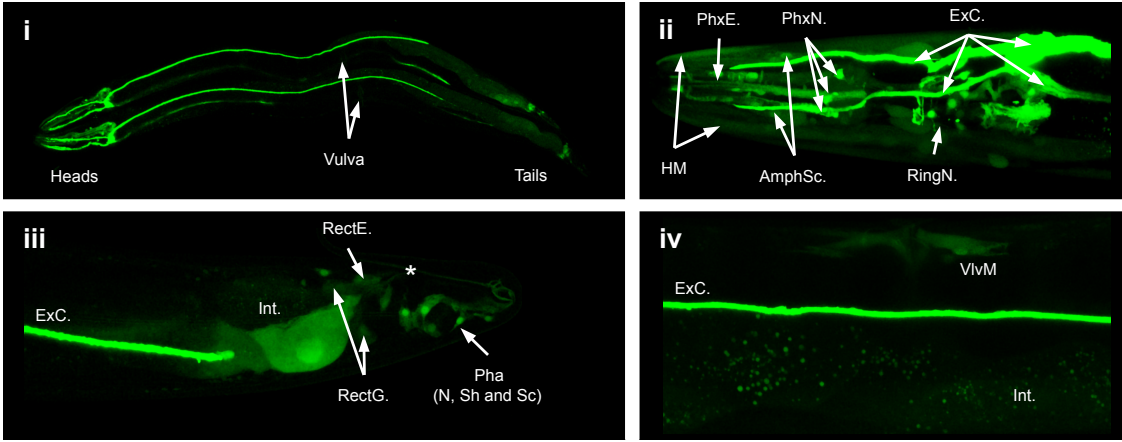

B

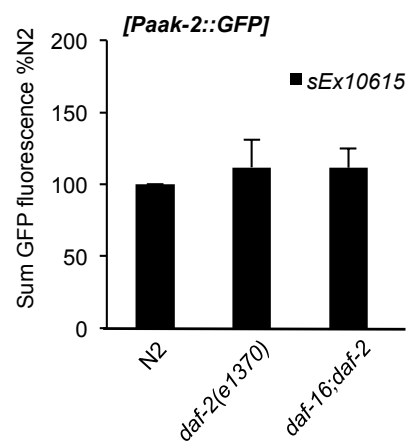

C

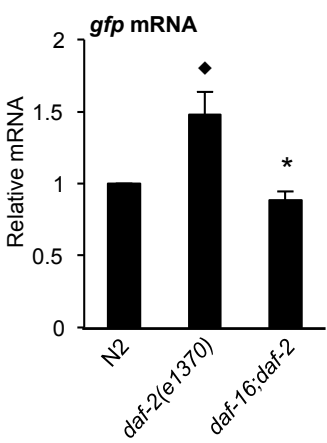

Supplement: Figure S8 — Evidence of regulation of aak-2 by insulin/IGF-1 signaling. A) Confocal images showing Paak-2::GFP expression pattern in 1-day old wild type hermaphrodites. The Paak-2::gfp sequence in sEx10615 contains 2.821 Kb of DNA upstream of the transcriptional start site. (i) Whole worm expression pattern. (ii) Head Paak-2::GFP expression is mostly seen in the excretory cell (Exc) but is also observed in pharyngeal neurons (PhxN), epithelial cells (PhxE), a subset of ring neurons (RingN), amphid socket cells (AmphSc) and head body wall muscles (HM). (iii) Paak-2::GFP is also expressed in the posterior intestine (Int), rectal gland (RectG) and epithelial cells (RectE), and in phasmids (Pha). (iv) Paak-2::GFP is detected in vulval muscles (VlvM). This expression pattern builds on but is consistent with previous observations of this strain [51] and another previous study [80] which used a Paak-2::aak-2::gfp translational reporter. Table S10 compares expression of Paak-2::gfp with other AMPK subunits. B) Quantification of GFP fluorescence in worms expressing a Paak-2::GFP reporter. We did not observe any differences in GFP fluorescence levels when our transgene was crossed into daf-2 or daf-16; daf-2 backgrounds. The expression pattern was also unchanged (data not shown). GFP fluorescence values are corrected for background intestinal autofluorescence using values obtained from non-transgenic animals (see methods). Error bars, standard deviation. C) qRT-PCR of gfp mRNA in worms expressing a Paak-2::GFP reporter revealed a small, significant change in expression between daf-2 and daf-2; daf-16. ♦p<0.05 compared to N2, *p<0.05 compared to daf-2. Error bars, standard deviation. (PDF) [file pgen.1004109.s008.pdf]

Figure S9

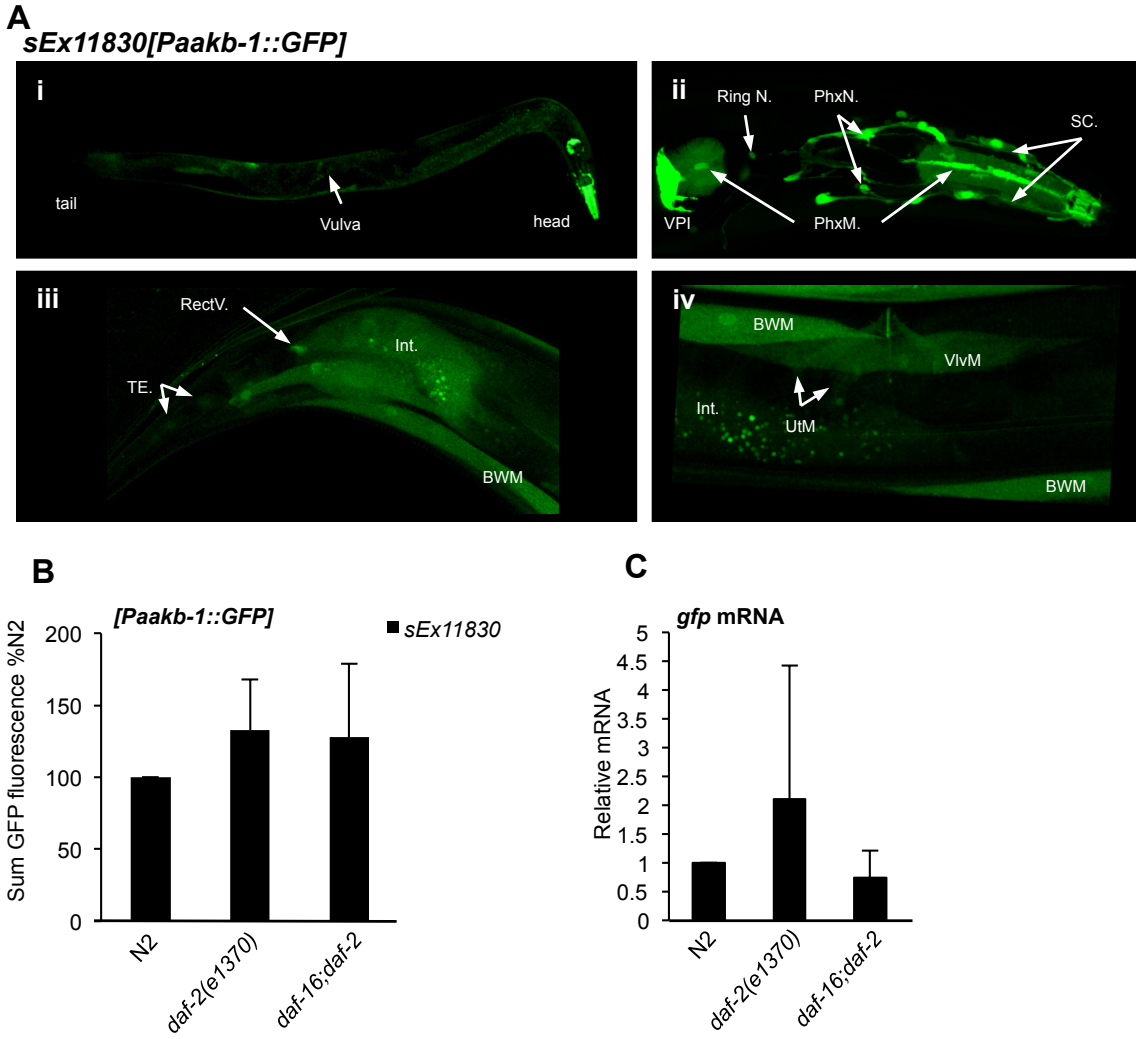

Supplement: Figure S9 — Expression pattern of aakb-1. A) Confocal images showing Paakb-1::GFP in 1-day old wild type hermaphrodites. The Paakb-1::gfp reporter contains 2.845 Kb of promoter directly upstream of the transcriptional start site. (i) Whole worm expression pattern. (ii) Paakb-1::GFP is strongly expressed in pharyngeal muscles (PhxM), the pharyngeal intestinal valve cells (VPI) as previously noted [51] and a few head neurons of the pharynx (PhxN) and the ring region (RingN). It is also detected in some support cells (SC). (iii) Tail expression of Paakb-1::GFP includes the tail minor epithelium (TE), the rectal-intestinal valve (RectV), posterior intestine and body wall muscles (BWM). (iv) Mid-body expression is mostly restricted to muscles of the vulva (VlvM), uterus (UtM) and body wall (BMW), but is also detected in the intestine (Int). However we noted that the levels of GFP expression in all genetic backgrounds was highly variable between animals. Table S10 compares expression of Paakb-1::gfp with other AMPK subunits. B) Quantification of GFP fluorescence in the heads of worms expressing a Paakb-1::GFP reporter. The daf-16; daf-2 background carrying this transgene exhibited unexplained and higher than normal intestinal autofluorescence making fluorescence quantification of whole worms difficult. To circumvent this we measured GFP fluorescence levels in the heads of worms, where Paakb-1::GFP is highly expressed. Mean of four trials shown. Error bars, standard deviation. C) qRT-PCR of gfp mRNA in worms expressing a Paakb-1::GFP reporter revealed an increase in expression between daf-2 and daf-2; daf-16, however this was not significant, probably due to transgene expression varying between individuals. Error bars, standard deviation. (PDF) [file pgen.1004109.s009.pdf]

**Figure S10**

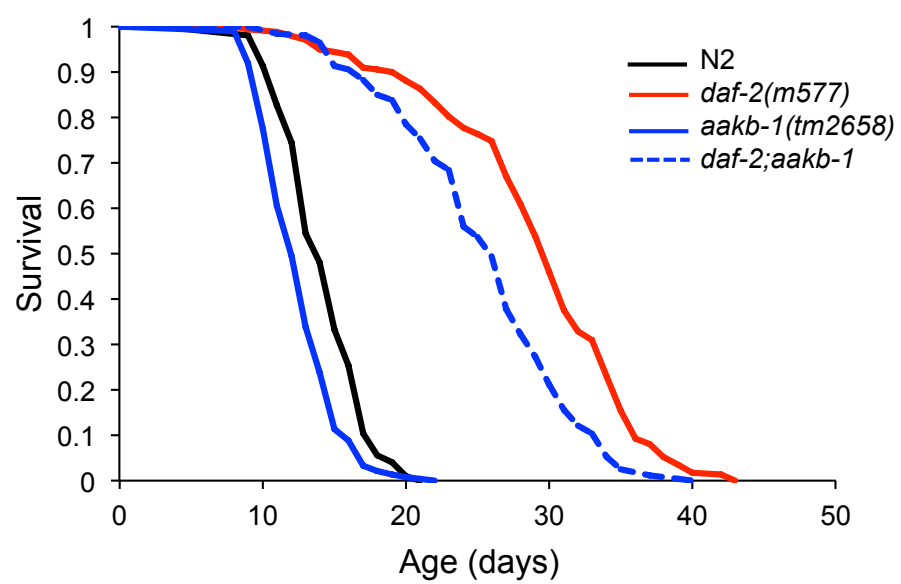

Supplement: Figure S10 — aakb-1 does not contribute to daf-2 longevity. The proportional reduction in lifespan caused by aakb-1(tm2658) does not significantly differ between daf-2(+) and daf-2(m577) backgrounds. The combined data from 4 trials is shown (C in Table S6). (PDF) [file pgen.1004109.s010.pdf]

**Figure S11**

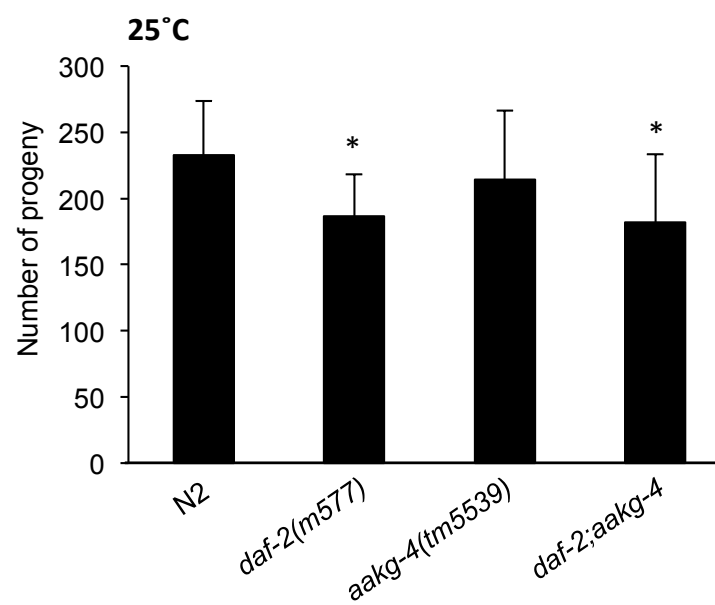

Supplement: Figure S11 — aakg-4(tm5539) does not alter progeny production. The total progeny were counted from individual adult worms. This graph represents data from two independent trials. n = 20 broods for each genotype, * p<0.05 compared to N2. (PDF) [file pgen.1004109.s011.pdf]

**Figure S12**

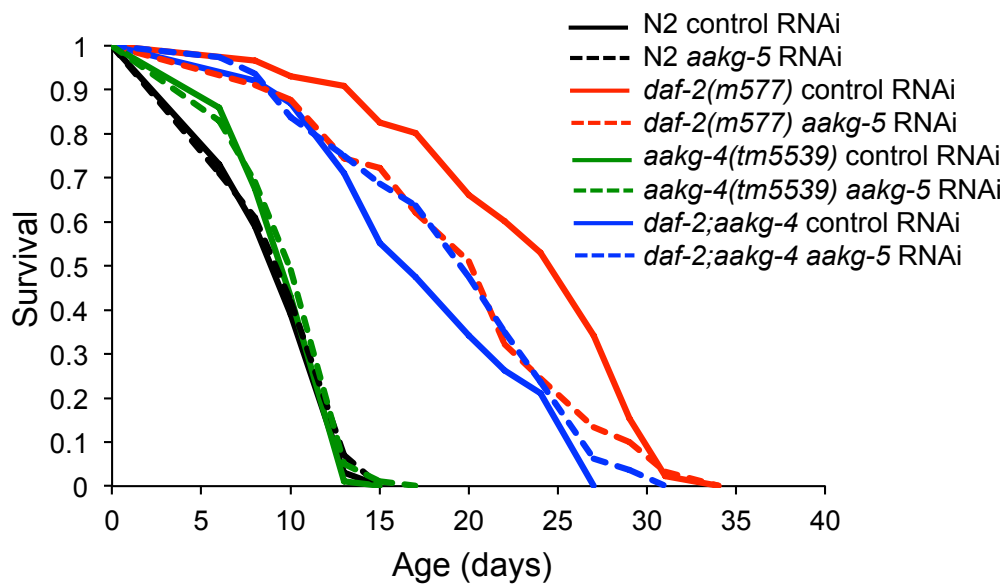

Supplement: Figure S12 — aakg-5 RNAi shortens daf-2 lifespan. One representative experiment is shown corresponding to trial 9 in Table S8. Reduction of aakg-5 mRNA was confirmed by qRT-PCR (data not shown). However, aakg-5 RNAi did not further reduce the lifespan of daf-2(m577);aakg-4(tm5539) double mutants. (PDF) [file pgen.1004109.s012.pdf]

**Figure S13**

**A**

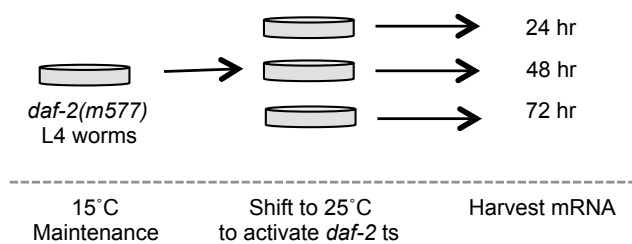

**B**

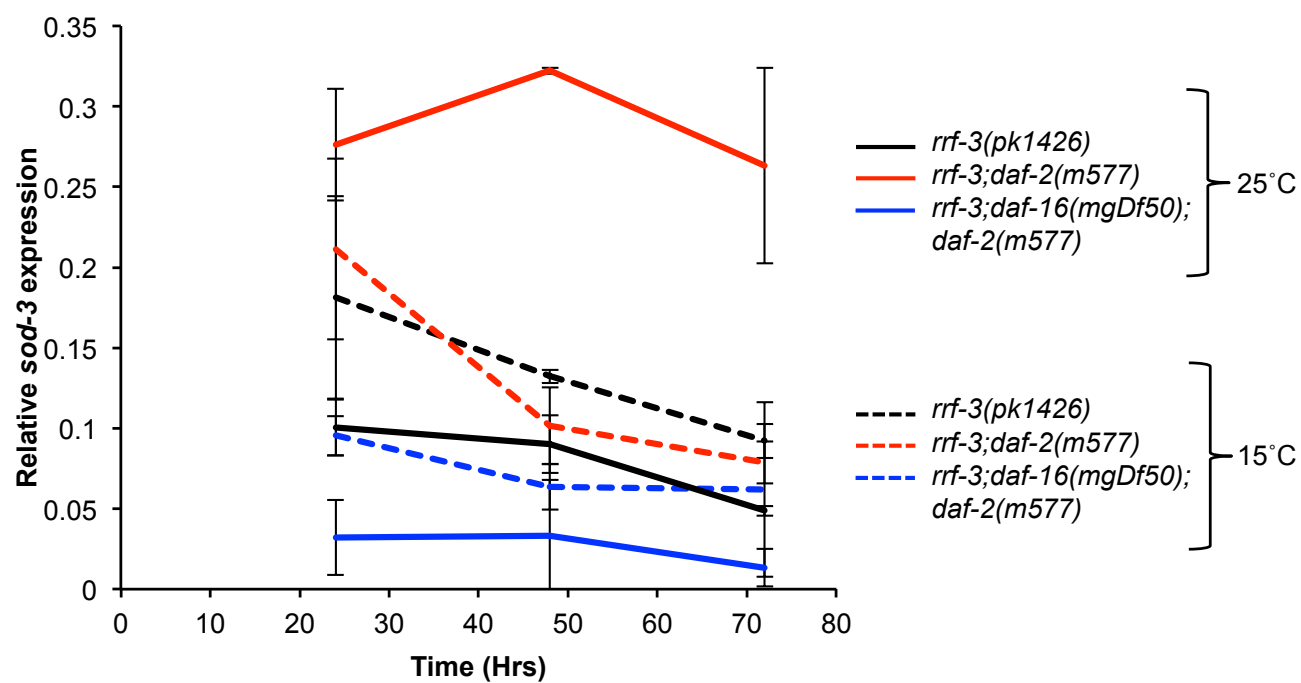

Supplement: Figure S13 — Induction of the DAF-16 target gene sod-3 in daf-2 mutants. A) L4 daf-2(m577) animals were shifted to 25°C prior to RNA extraction. B) sod-3 mRNA levels are induced in response to temperature specifically in daf-2 mutants. (PDF) [file pgen.1004109.s013.pdf]

Figure S14

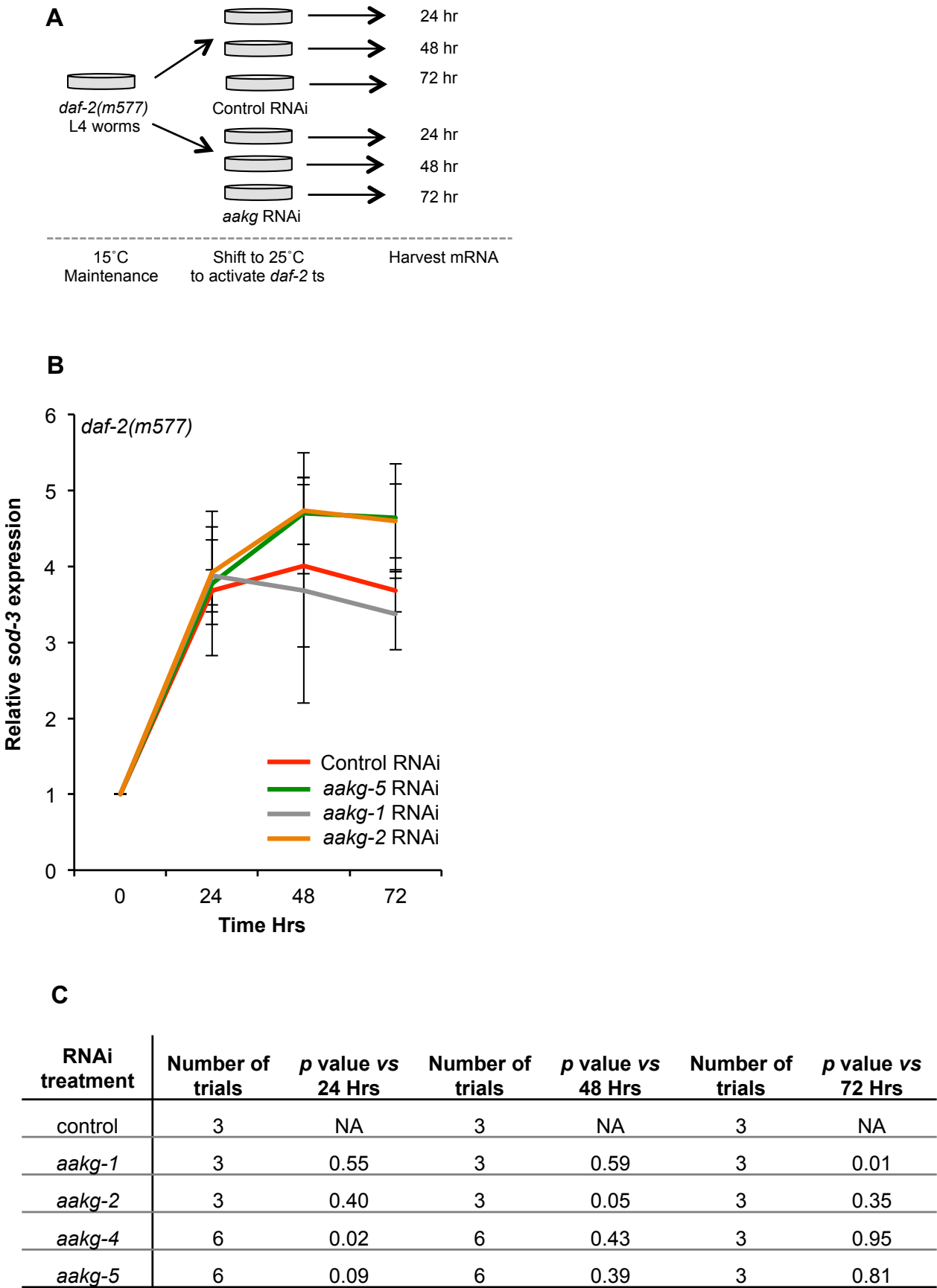

Supplement: Figure S14 — Effect of aakg RNAi on induction of sod-3 expression by reduced IIS. A) Experimental protocol. L4 daf-2(m577) animals were treated with aakg RNAi and shifted to 25°C prior to RNA extraction (Figure 4A). B) sod-3 mRNA levels are induced in response to temperature but this induction is not reduced by aakg-1, aakg-2 or aakg-5 RNAi. The level of sod-3 at 0 hr is that of adults that have not been shifted to 25°C i.e. maintained at 15°C. Error bars, standard deviation. These RNAi treatments were carried out in parallel with aakg-4 RNAi (Figure 4B). C) Statistical analysis of data generated on pooled data sets for each time point and RNAi treatment. (PDF) [file pgen.1004109.s014.pdf]

**Figure S15**

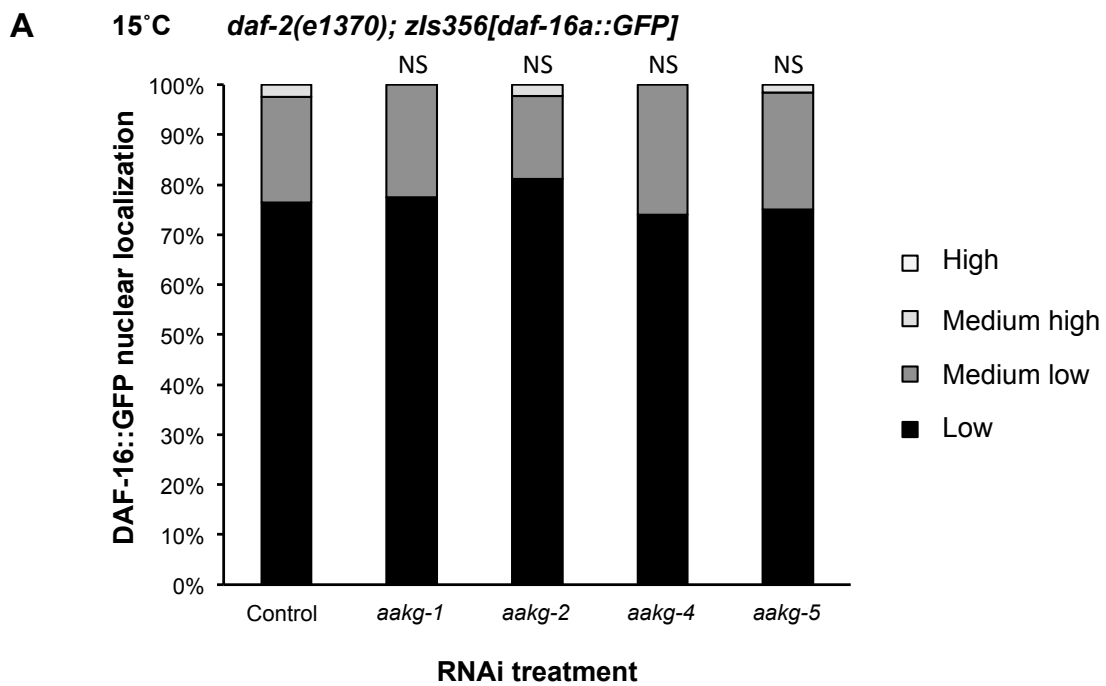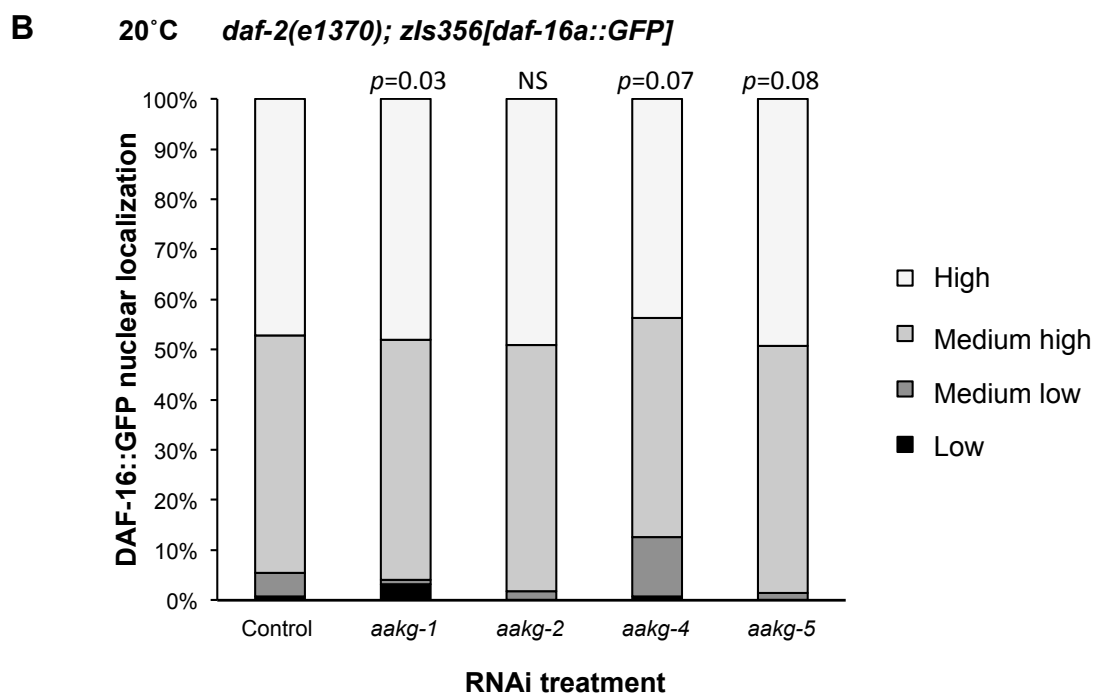

Supplement: Figure S15 — Effects of aakg RNAi on DAF-16::GFP nuclear localization. We created a strain that expressed the DAF-16a::GFP transgene [81] in a daf-2(e1370) background. daf-2(e1370) is a particularly strong class 2 daf-2 allele, and DAF-16::GFP shows exclusively nuclear localization at 25°C. However, at A) 15°C or B) 20°C (temperatures where this allele still extends lifespan), weaker DAF-16::GFP nuclear localization is seen [82]. 70–100 animals (per group) were scored for DAF-16::GFP nuclear localization using a scoring system similar to that used in [83]. p, Chi Squared test vs control. (PDF) [file pgen.1004109.s015.pdf]

Figure S16

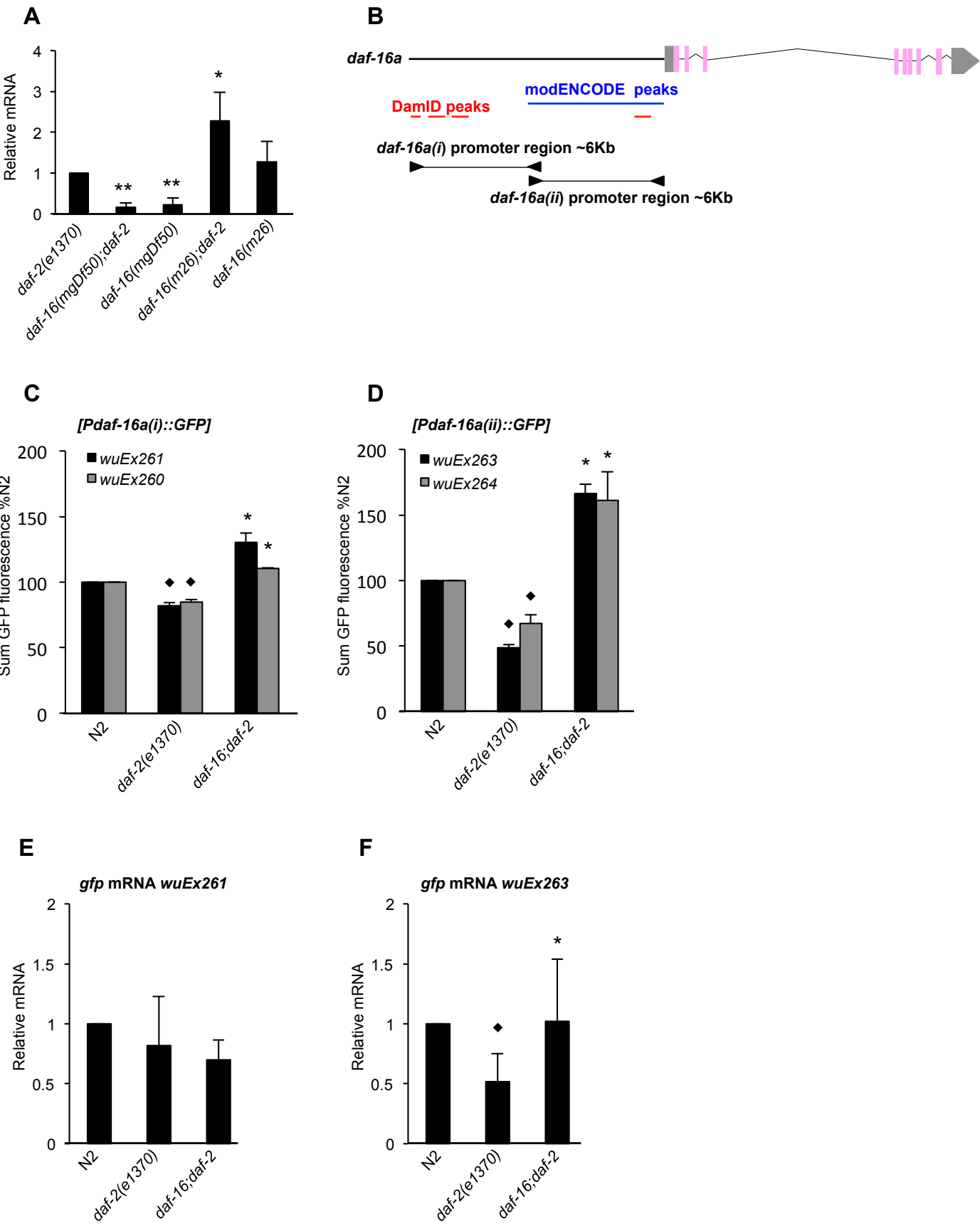

Supplement: Figure S16 — DAF-16 does not regulate its own transcription. A) qRT-PCR shows that daf-16 mRNA levels are affected in a daf-2 background by daf-16(mgDf50) (a deletion allele) but not by daf-16(m26) (a point mutant allele). **p<0.002, *p = 0.03. B) It remains possible that daf-16 activates its own expression in some worm tissues or cell types, but that this is not detectable in whole worm mRNA extracts. To test this we created and examined Pdaf-16a::gfp transcriptional reporters. Given the large size of the predicted DAF-16-binding region, 2 types of Pdaf-16a::gfp reporters, i and ii, were created containing overlapping 5′ and 3′ regions of the promoter daf-16a::GFP transgenes were designed based on DAF-16 binding sites predicted by DamID and modENCODE. Transgenes were introduced as extrachromosomal arrays into wild-type worms and two independent transgenic lines for each construct were isolated. In a wild-type background, both constructs led to near ubiquitous GFP expression patterns similar to those previously reported for DAF-16a::GFP translational reporters [6], [53], [81]. C, D) Quantification of GFP fluorescence in worms expressing Pdaf-16a(i)::GFP or Pdaf-16a(ii)::GFP reporters. We observed a decrease in GFP fluorescence levels when our transgene was crossed into daf-2 which was reversed by daf-16. The same was also true for a second set of strains generated from different extrachromosomal arrays. The expression pattern was unchanged (data not shown). ♦ p<0.01 compared to N2 *p<0.01 compared to daf-2. Error bars, standard deviation. E, F) Quantification of gfp mRNA in worms expressing Pdaf-16a(i)::GFP or Pdaf-16a(ii)::GFP reporters. ♦ p<0.01 compared to N2, *p<0.01 compared to daf-2. Error bars, standard deviation. In worms with Pdaf-16a(i)::gfp reporters, GFP fluorescence levels were slightly decreased in daf-2(e1370) compared to daf-16(mgDf50); daf-2 backgrounds, but gfp mRNA levels were not (Figure S16C and S16E). However, in worms with Pdaf-16a(ii)::gfp reporters, GFP [file pgen.1004109.s016.pdf]
