# Supplementary material for: DAF-16/FoxO Directly Regulates an Atypical AMP-Activated Protein Kinase Gamma Isoform to Mediate the Effects of Insulin/IGF-1 Signaling on Aging in Caenorhabditis elegans
Source: PLoS Genet. 2014 Feb 6;10(2):e1004109. doi: 10.1371/journal.pgen.1004109 (PMC3916255; doi:10.1371/journal.pgen.1004109)
Supplement: Figure S3 — Evolutionary relationship between nematode and platyhelminth lineages. Species shown are represented in Figure 1A, S1 and S2. (PDF) [file pgen.1004109.s003.pdf]

Figure S3

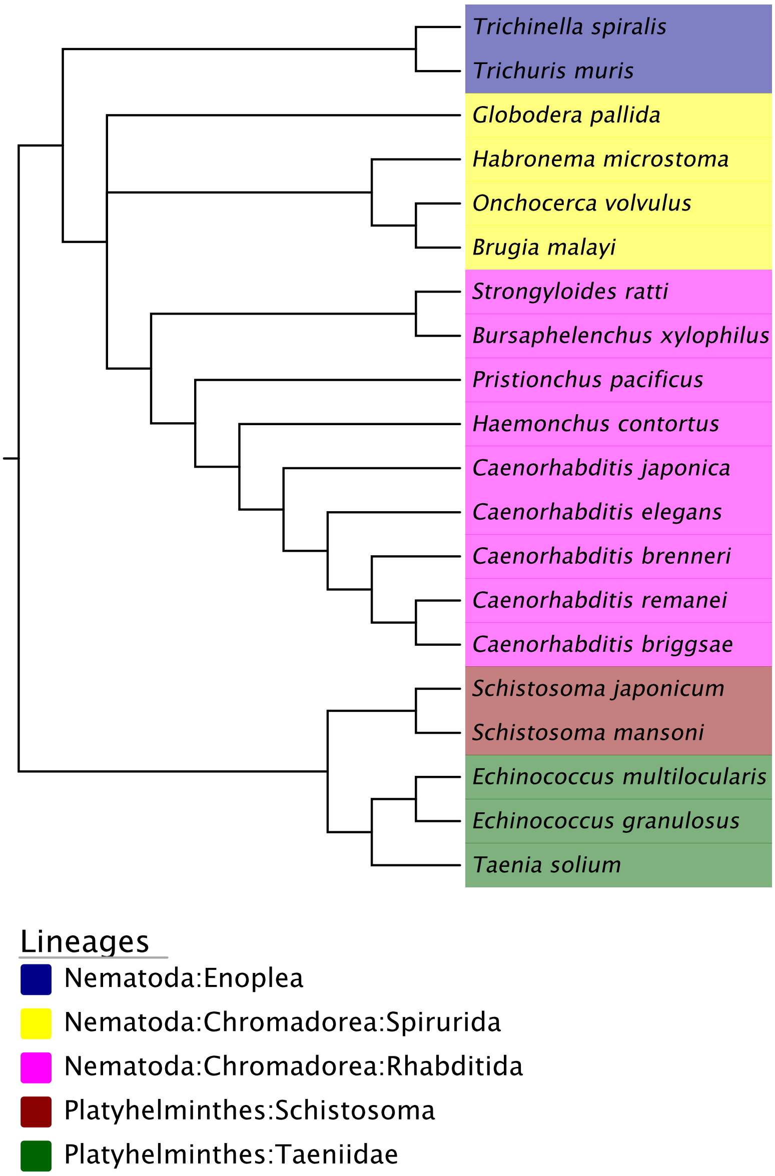
Trichinella spiralis
Trichuris muris
Globodera pallida
Habronema microstoma
Onchocerca volvulus
Brugia malayi
Strongyloides ratti
Bursaphelenchus xylophilus
Pristionchus pacificus
Haemonchus contortus
Caenorhabditis japonica
Caenorhabditis elegans
Caenorhabditis brenneri
Caenorhabditis remanei
Caenorhabditis briggsae
Schistosoma japonicum
Schistosoma mansoni
Echinococcus multilocularis
Echinococcus granulosus
Taenia solium
Lineages
Nematoda:Enoplea
Nematoda:Chromadorea:Spirurida
Nematoda:Chromadorea:Rhabditida
Platyhelminthes:Schistosoma
Platyhelminthes:Taeniidae
