# Supplementary material for: DAF-16/FoxO Directly Regulates an Atypical AMP-Activated Protein Kinase Gamma Isoform to Mediate the Effects of Insulin/IGF-1 Signaling on Aging in Caenorhabditis elegans
Source: PLoS Genet. 2014 Feb 6;10(2):e1004109. doi: 10.1371/journal.pgen.1004109 (PMC3916255; doi:10.1371/journal.pgen.1004109)
Supplement: Table S1 — Percentage amino acid sequence similarity of C. elegans AMPK γ subunit CBS domains to human PRKAG1. (PDF) [file pgen.1004109.s017.pdf]

Gene	CBS Domain 1	2	3	4
aakg-1	65%	66%	70%	74%
aakg-2	56%	70%	60%	64%
aakg-3	62%	60%	51%	71%
aakg-4	10%	50%	43%	48%
aakg-5	12%	52%	57%	50%

Table S1. Percentage amino acid sequence similarity of *C. elegans* AMPK γ subunit CBS domains to human PRKAG1.
