# Supplementary material for: DAF-16/FoxO Directly Regulates an Atypical AMP-Activated Protein Kinase Gamma Isoform to Mediate the Effects of Insulin/IGF-1 Signaling on Aging in Caenorhabditis elegans
Source: PLoS Genet. 2014 Feb 6;10(2):e1004109. doi: 10.1371/journal.pgen.1004109 (PMC3916255; doi:10.1371/journal.pgen.1004109)
Supplement: Table S2 — AMP/ATP binding residues in Rhabditida. All sequences obtained were aligned using ClustalW with an identity matrix. Residues known to be involved in nucleotide binding were identified using the human PRKAG1 gene. Atypical residues are in red. Grey blocks show sequences that cluster with C. elegans atypical isoforms aakg-4 and aakg-5 (Figure S2). (PDF) [file pgen.1004109.s018.pdf]

	Residue (Human *PRKAG1* numbering)				
**Species and gene**	**70**	**150**	**151**	**298**	**299**
Human *PRKAG1*	R	H	R	H	R
C. elegans aakg-1	R	H	R	H	R
C. elegans aakg-2	R	H	R	H	R
C. elegans aakg-3	R	H	R	H	R
C. elegans aakg-4	I	S	F	S	S
C. elegans aakg-5	T	H	R	Q	S
C. briggsae aakg-1 CBG11682	R	H	R	H	R
C. briggsae aakg-2	R	H	R	H	R
C. briggsae aakg-3	R	H	R	H	R
C. briggsae aakg-4	I	S	F	C	C
C. briggsae aakg-5	T	H	R	Q	S
C. remanei CRE19909	R	H	R	H	R
C. remanei aakg-2	R	H	R	H	R
C. remanei aakg-3	R	H	R	H	R
C. remanei aakg-4	I	S	F	S	C
C. remanei aakg-5	T	H	R	Q	S
C. remanei CRE19910	-	-	-	-	-
C. brenneri CBN05184	R	R	H	H	R
C. brenneri Cbn-aakg-2	R	H	R	H	R
C. brenneri Cbn-aakg-3	R	H	R	H	R
C. brenneri Cbn-aakg-4	I	S	F	S	S
C. brenneri CBN19031	T	H	R	Q	S
C. japonica CJA14721a	R	H	R	H	R
C. japonica Cjp-aakg-2	R	H	R	H	R
C. japonica Cjp-aakg-3	-	-	-	H	R
C. japonica Cjp aakg-4	I	S	F	N	C
C. japonica CJA32986b	R	-	-	-	-
P. pacificus 1	R	H	R	-	-
P. pacificus 2	R	H	R	H	R
P. pacificus 3	R	H	R	Y	R
P. pacificus 4	S	H	R	-	-
P. pacificus 5	G	-	-	-	-
H. contortus 00980100.t1 1	R	H	R	H	R
H. contortus 02057000.t2 1	R	H	R	H	R
H. contortus 00002900.t1 1	R	H	R	Q	R
H. contortus 01835300.t1 1	R	H	R	M	R
H. contortus 00949100.t3 1	Q	H	R	-	-

Table S2. AMP/ATP binding residues in Rhabditida.
