# Supplementary material for: DAF-16/FoxO Directly Regulates an Atypical AMP-Activated Protein Kinase Gamma Isoform to Mediate the Effects of Insulin/IGF-1 Signaling on Aging in Caenorhabditis elegans
Source: PLoS Genet. 2014 Feb 6;10(2):e1004109. doi: 10.1371/journal.pgen.1004109 (PMC3916255; doi:10.1371/journal.pgen.1004109)
Supplement: Table S3 — AMP/ATP binding residues in Nematoda and Platyhelminthes. All sequences obtained were aligned simultaneously using ClustalW with an identity matrix. Residues known to be involved in nucleotide binding were identified using the human PRKAG1 gene. Atypical residues are in red. Grey blocks show isoforms that cluster with C. elegans atypical aakg-4 and aakg-5 which all contain atypical residues. A number of the γ subunit isoforms from various more distant species contain missing or truncated cystathione-β-synthase (CBS) domains, it is possible that these may be pseudogenes. Alternatively, in AMPK complexes containing these isoforms, enzyme function may be compromised by lacking these CBS domains. However, it is possible that only two CBS domains can come together to form the nucleotide-binding region, as seen in other CBS-domain containing proteins [84]. It remains to be seen whether in this situation the absence of conserved nucleotide-interacting residues would allow the enzyme to respond to changes in the AMP/ATP ratio. (PDF) [file pgen.1004109.s019.pdf]

Species and gene	Residue (Human *PRKAG1* numbering) 70	150	151	298	299	
T. spiralis 003373079.1	R	H	R	H	R	Nematoda
T. spiralis 003373447.1	L	H	R	H	R	
T. muris 1	R	H	R	-	-	
T. muris 2	-	-	-	H	R	
G. pallida 001103800	R	H	R	S	E	
H. microstoma_000072800.1	R	H	R	H	R	
H. microstoma_000177500.1	S	T	T	S	C	
H. microstoma_000994900.1	R	H	R	H	R	
O. volvulus_665_aa_scaffold2.1	R	H	R	H	R	
O. volvulus_272_aa_scaffold146.1	-	H	R	H	R	
B. malayi 1	R	H	R	H	R	
B. malayi 2	-	-	-	H	R	
B. malayi 4	E	H	R	D	Q	
B. malayi 5	-	-	-	-	-	
S. ratti 321 020524600.t1	R	-	-	H	R	
S. ratti 321 020553100.t1	Q	K	R	N	A	
B. xylophilus s00333.119.1	R	H	R	H	R	
B. xylophilus s00579.530.1	R	H	R	S	Q	
S. japonicum_0031410.1	-	H	R	-	-	Platyhelminthes
S. japonicum_0023410.1	R	H	R	-	-	
S. japonicum_0030130.1	R	-	-	-	-	
S. mansoni 127420.1	R	H	H	R	S	
S. mansoni 142030.1	-	-	-	H	R	
S. mansoni 144700.1	M	P	Y	S	A	
E. multilocularis_001024900.1	R	H	R	H	R	
E. multilocularis_001057300.1	R	H	R	H	R	
E. granulosus_001024900.1	R	H	R	H	R	
E. granulosus_001057300.1	R	H	R	H	R	
T. solium_000191400.1	R	H	R	H	R	
T. solium_000222200.1	R	H	R	-	-	

Table S3. AMP/ATP binding residues in Nematoda and Platyhelminthes.
