# Supplementary material for: DAF-16/FoxO Directly Regulates an Atypical AMP-Activated Protein Kinase Gamma Isoform to Mediate the Effects of Insulin/IGF-1 Signaling on Aging in Caenorhabditis elegans
Source: PLoS Genet. 2014 Feb 6;10(2):e1004109. doi: 10.1371/journal.pgen.1004109 (PMC3916255; doi:10.1371/journal.pgen.1004109)
Supplement: Table S4 — Effects of insulin/IGF-1 signaling on AMPK gene expression. Fold changes in mRNA levels are shown. Some p values are adjusted and some are not, those in bold are considered significant by the authors. For studies comparing daf-2 with daf-16(mgDf50);daf-2 values are not noted for daf-16 as probe sets fall within the boundaries of the mgDf50 deletion. McElwee et al., 2003: the numbers are an average of four replicates and SD = standard deviation. Budovskaya et al., 2008: negative numbers indicate that the gene is positively regulated by DAF-16. Values represent a total difference across Stanford and Caltech arrays. No differences in aak-1, aakb-1 or aakg-1-3 mRNA levels were detected by any of these studies. (PDF) [file pgen.1004109.s020.pdf]

**Study**	**Comparison**	***daf-16***	**p**	***aak-2***	**p**	***aakb-1***	**p**	***aakg-4***	**p**	***aakg-5***	***p***
McElwee *et al*., 2003 (reanalyzed by McElwee *et al.,* 2007)	*glp-4(bn2); daf-2(e1370) vs daf-16(m27); glp-4(bn2); daf-2(e1370)*	**1.84**	**SD 0.20**	1.06	SD 0.23	**2.14**	**SD 1.70**	**6.72**	**SD 0.24**	**6.06**	**SD 1.35**
McElwee *et al*., 2004	*daf-2(e1370) vs daf-16(mgDf50); daf-2(e1370)*			**1.84**	**0.12**	**3.68**	**0.02**	**8.40**	**0.00**	**2.79**	**0.09**
McElwee *et al*., 2004	*daf-2(m577) vs daf-16(mgDf50); daf-2(m577)*			**2.28**	**0.00**	**7.51**	**0.00**	**6.63**	**0.00**	**3.53**	**0.00**
Budovskaya *et al*., 2008	*fer-15(b26); daf-16(m26) vs fer-15(b26)*	**-0.942**<br>-0.171	**0**<br>0.19	0.02	0.94	**-0.50**	**0**	**-0.47**	**0.00**	-0.02	0.95
Golden and Melov 2004	*daf-2(e1370) vs* N2 (single worms at 19 days)					**0.63**	**0.00**				
Troemel *et al*., 2006	*daf-2(e1370) vs daf-16(mgDf50); daf-2(e1370)*			-0.283	0.46	0.153	0.75	0.496	0.09	-0.160	0.49
qRT-PCR (This study)	N2 *vs daf-2(e1370)*			+2.52	0.30	+3.00	0.08	**+5.26**	**0.01**	+1.49	0.39
	daf-2(e1370) vs daf-16(mgDf50); daf-2(e1370)			-1.58	0.50	-3.03	0.08	**-9.15**	**0.01**	-0.92	0.87

Table S4. Effects of insulin/IGF-1 signaling on AMPK gene expression.
