# Supplementary material for: DAF-16/FoxO Directly Regulates an Atypical AMP-Activated Protein Kinase Gamma Isoform to Mediate the Effects of Insulin/IGF-1 Signaling on Aging in Caenorhabditis elegans
Source: PLoS Genet. 2014 Feb 6;10(2):e1004109. doi: 10.1371/journal.pgen.1004109 (PMC3916255; doi:10.1371/journal.pgen.1004109)
Supplement: Table S5 — Statistical analysis of ChIP data. The % input for three individual trials was compared for the two aakg-4 peaks and one aakg-5 peak (Figure S5). aakg-4 peak 2 and aakg-5 peak 1 were in the promoter fragments that we examined in more detail. Only the aakg-4 peak contains DAF-16 binding elements. Each experiment was analysed individually and then the data sets combined and tested for significant differences using a mixed effects linear model (MELM). The values obtained for a positive control DAF-16 target gene M01H9.3 [22] are also shown. (PDF) [file pgen.1004109.s021.pdf]

Gene	DamID peak	% input *daf-2(e1370)* compared to % input *daf-16(mgDf50);daf-2* *p* value (ttest)			% input *daf-2(e1370)* compared to % input *daf-16;daf-2* *p* value (MELM)
		**Trial 1**	**Trial 2**	**Trial 3**	
aakg-4	1	NS (0.06)	0.04	NS	NS (0.08)
aakg-4	2	0.02	0.005	NS	0.01
aakg-5	1	NS	0.01	NS	NS (0.07)
M01H9.3		<0.001	0.02	0.003	<0.0001

Table S5. Statistical analysis of ChIP data.
