# Supplementary material for: DAF-16/FoxO Directly Regulates an Atypical AMP-Activated Protein Kinase Gamma Isoform to Mediate the Effects of Insulin/IGF-1 Signaling on Aging in Caenorhabditis elegans
Source: PLoS Genet. 2014 Feb 6;10(2):e1004109. doi: 10.1371/journal.pgen.1004109 (PMC3916255; doi:10.1371/journal.pgen.1004109)
Supplement: Table S6 — Effects of aakb-1(tm2658) on lifespan. aakb-1(tm2658) shortens lifespan modestly and to a similar degree in daf-2(+) and daf-2(−) backgrounds. This argues against a role for increased aakb-1 expression as a cause of daf-2 Age. C, data from all four trials combined. N, number of worms (total worms scored including those censored). Lifespan assays all carried out at 25°C. p, Probability (determined by log rank test) of being the same as a N2 or b daf-2(m577). % difference in mean lifespan compared to a N2 or b daf-2(m577). SEM, standard error of the mean. (PDF) [file pgen.1004109.s022.pdf]

Strain	Genotype	Trial	Mean lifespan ± SEM (days)	% Difference	N (total)	*p*
	N2	C	14.3 ± 0.14		379 (384)	
GA1061	*aakb-1(tm2658)*	C	12.6 ± 0.15	-13.00	283 (291)	<0.0001[a]
DR1567	*daf-2(m577)*	C	29.1 ± 0.37		324 (351)	
GA1069	*aakb-1; daf-2*	C	25.5 ± 0.32	-14.17	325 (403)	<0.0001[a/b]
	N2	1	14.2 ± 0.25		82 (84)	
GA1061	*aakb-1(tm2658)*	1	12.5 ± 0.23	-12.0 [a]	94 (99)	<0.0001[a]
DR1567	*daf-2(m577)*	1	31.2 ± 0.84		59 (76)	<0.0001[a]
GA1069	*aakb-1; daf-2*	1	24.2 ± 0.54	-22.0 [b]	78 (80)	<0.0001[a/b]
	N2	2	13.5 ± 0.28		97 (100)	
GA1061	*aakb-1*	2	13.1 ± 0.29	-3 [a]	72 (75)	NS [a]
DR1567	*daf-2*	2	29.8 ± 0.97		51 (52)	<0.0001[a]
GA1069	*aakb-1; daf-2*	2	27.2 ± 0.59	-8.6 [b]	61 (71)	<0.0001[a/b]
	N2	3	14.4 ± 0.30		100 (100)	
GA1061	*aakb-1*	3	12.6 ± 0.37	-12.0 [a]	50 (50)	0.0002 [a]
DR1567	*daf-2*	3	28.7 ± 0.65		116 (122)	<0.0001[a]
GA1069	*aakb-1; daf-2*	3	25.9 ± 0.70	-9.5 [b]	97 (126)	<0.0001[a]<br>0.0036 [b]
	N2	4	15.1 ± 0.24		100 (100)	
GA1061	*aakb-1*	4	12.5 ± 0.36	-17.2 [a]	66 (67)	<0.0001[a]
DR1567	*daf-2*	4	28.0 ± 0.61		98 (101)	<0.0001[a]
GA1069	*aakb-1; daf-2*	4	25.2 ± 0.61	-10.0 [b]	88 (126)	<0.0001[a]<br>0.0004 [b]

Table S6. Effects of *aakb-1(tm2658)* on lifespan.
