# Supplementary material for: DAF-16/FoxO Directly Regulates an Atypical AMP-Activated Protein Kinase Gamma Isoform to Mediate the Effects of Insulin/IGF-1 Signaling on Aging in Caenorhabditis elegans
Source: PLoS Genet. 2014 Feb 6;10(2):e1004109. doi: 10.1371/journal.pgen.1004109 (PMC3916255; doi:10.1371/journal.pgen.1004109)
Supplement: Table S7 — aakg-4(tm5539) shortens daf-2 lifespan. N, number of worms (Total worms scored including those censored). p, Probability (determined by log rank test) of being the same as a daf-2(m577), b N2 or c aakg-4(tm5539). % difference in mean lifespan compared to daf-2(m577). SEM, standard error of the mean. (PDF) [file pgen.1004109.s023.pdf]

Strain	Genotype	Trial	Mean lifespan ± SEM (Days)	% Difference	N (total)	*p*
	N2	1	11.1 ± 0.17		127 (127)	<0.0001 [a]
GA1071	*aakg-4(tm5539)*	1	11.2 ± 0.14		110 (123)	<0.0001 [a]<br>NS [b]
DR1567	*daf-2(m577)*	1	29.5 ± 0.59		115 (116)	-
GA1072	*daf-2(m577);aakg-4(tm5539)*	1	23.9 ± 0.48	-23.06	123 (123)	<0.0001 [a]
GA1001	*aak-2(ok524)*	1	9.7 ± 0.12		125 (125)	<0.0001 [c]
	N2	2	11.8 ± 0.18		121 (125)	<0.0001 [a]
GA1071	*aakg-4(tm5539)*	2	12.4 ± 0.23		102 (123)	<0.0001 [a]<br>0.0266 [b]
DR1567	*daf-2(m577)*	2	28.9 ± 0.45		95 (97)	-
GA1072	*daf-2(m577);aakg-4(tm5539)*	2	24.0 ± 0.41	-20.33	106 (110)	<0.0001 [a]
GA1001	*aak-2(ok524)*	2	9.3 ± 0.14		121 (121)	<0.0001 [c]
	N2	3	11.7 ± 0.13		118 (120)	<0.0001[a]
DR1567	*daf-2(m577)*	3	29.9 ± 0.50		110 (110)	-
GA1072	*daf-2(m577);aakg-4(tm5539)*	3	23.7 ± 0.36	-26.33	187 (187)	<0.0001[a]
	N2	4	11.3 ± 0.17		127 (127)	<0.0001[a]
DR1567	*daf-2(m577)*	4	29.0 ± 0.69		121 (124)	-
GA1072	*daf-2(m577);aakg-4(tm5539)*	4	22.7 ± 0.36	-27.85	241 (241)	<0.0001[a]

Table S7. *aakg-4(tm5539)* shortens *daf-2* lifespan.
