# Supplementary material for: DAF-16/FoxO Directly Regulates an Atypical AMP-Activated Protein Kinase Gamma Isoform to Mediate the Effects of Insulin/IGF-1 Signaling on Aging in Caenorhabditis elegans
Source: PLoS Genet. 2014 Feb 6;10(2):e1004109. doi: 10.1371/journal.pgen.1004109 (PMC3916255; doi:10.1371/journal.pgen.1004109)
Supplement: Table S8 — aakg-4 and aakg-5 RNAi shorten daf-2 lifespan. N, number of worms (total worms scored including those censored). The rrf-3 mutation sensitizes the animals to the effects of RNAi [52]. Lifespan assays all carried out at 25°C. It was confirmed by qRT-PCR that the appropriate aakg mRNA levels were reduced by aakg-4 or aakg-5(RNAi) (data not shown). In the case of aakg-5 RNAi we sometimes observed an additional decrease in aakg-4 mRNA levels in worms that had been fed RNAi for longer times suggesting that there may be some cross RNAi that could affect lifespan. p, probability (determined by log rank test) of being the same as: a rrf-3(pk1426); daf-2(m577) or daf-2(m577) control RNAi. b rrf-3(pk1426) or N2 control RNAi. c aakg-4(tm5539). d daf-2; aakg-4(tm5539). % difference in mean lifespan compared to a rrf-3(pk1426) or N2. b rrf-3(pk1426); daf-2(m577) or daf-2(m577). c aakg-4(tm5539). d daf-2; aakg-4(tm5539). SEM = standard error of the mean. NS, not significant (p>0.05). (PDF) [file pgen.1004109.s024.pdf]

Strain	Genotype and RNAi treatment	Trial	Mean lifespan ± SEM (days)	% Difference	N (total)	*p*
GA303	*rrf-3; daf-2* control RNAi	1	29.2 ± 0.98		73 (98)	-
GA303	*rrf-3; daf-2 aakg-4* RNAi	1	28.0 ± 0.72	-3.8 [b]	78 (96)	0.047 [a]
GA303	*rrf-3; daf-2 aakg-5* RNAi	1	27.5 ± 1.08	-5.6 [b]	56 (82)	NS [a]
NL2099	*rrf-3* control RNAi	2	14.8 ± 0.52		86 (99)	
GA303	*rrf-3; daf-2* control RNAi	2	28.4 ± 1.08		86 (88)	<0.0001[b]
GA303	*rrf-3; daf-2 aakg-4* RNAi	2	23.2 ± 0.79	-18.4 [b]	109 (109)	<0.0001[a]
GA303	*rrf-3; daf-2 aakg-5* RNAi	2	25.9 ± 0.99	-9.0 [b]	108 (108)	0.07 [a]
NL2099	*rrf-3* control RNAi	3	13.7 ± 0.47		82 (82)	-
GA303	*rrf-3; daf-2* control RNAi	3	26.5 ± 0.83		106 (118)	<0.0001[b]
GA303	*rrf-3; daf-2 aakg-4* RNAi	3	22.9 ± 0.70	-13.7 [b]	108 (108)	<0.0001[a]
GA303	*rrf-3; daf-2 aakg-5* RNAi	3	26.0 ± 1.10	-2.1 [b]	81 (81)	NS [a]
NL2099	*rrf-3* control RNAi	4	12.6 ± 0.34		119 (123)	-
NL2099	*rrf-3 aakg-4* RNAi	4	11.8 ± 0.35	-6.5 [a]	79 (81)	0.08 [b]
GA303	*rrf-3; daf-2* control RNAi	4	26.6 ± 0.86		127 (127)	<0.0001[b]
GA303	*rrf-3; daf-2 aakg-4* RNAi	4	20.0 ± 0.72	-24.8 [b]	102 (102)	<0.0001[a/b]
GA303	*rrf-3; daf-2 aakg-5* RNAi	4	17.8 ± 0.72	-32.9 [b]	114 (114)	<0.0001[a/b]
NL2099	*rrf-3* control RNAi	5	11.6 ± 0.39		49 (50)	-
NL2099	*rrf-3 aakg-4* RNAi	5	10.8 ± 0.36	-6.9 [a]	49 (50)	NS [b]
NL2099	*rrf-3 aakg-5* RNAi	5	10.9 ± 0.39	-5.9 [a]	50 (50)	NS [b]
GA303	*rrf-3; daf-2* control RNAi	5	27.9 ± 1.01		43 (50)	<0.0001[b]
GA303	*rrf-3; daf-2 aakg-4* RNAi	5	22.9 ± 0.53	-18.0 [b]	47 (50)	<0.0001[a/b]
GA303	*rrf-3; daf-2 aakg-5* RNAi	5	29.0 ± 0.72	+4.0 [b]	47 (50)	NS [a]<br><0.0001[b]
NL2099	*rrf-3* control RNAi	6	9.2 ± 0.27		75(75)	-
NL2099	*rrf-3 aakg-4* RNAi	6	10.3 ± 0.26	+11.8 [a]	73 (75)	0.005 [a]
GA303	*rrf-3; daf-2* control RNAi	6	27.1 ± 0.47		56 (60)	<0.0001[a]
GA303	*rrf-3; daf-2 aakg-4* RNAi	6	24.1 ± 0.52	-10.8 [b]	73 (75)	<0.0001[a]<br>0.0002[b]
NL2099	*rrf-3* control RNAi	7	12.1 ± 0.33		67 (67)	-
NL2099	*rrf-3 aakg-4* RNAi	7	11.0 ± 0.31	-8.9 [a]	66 (68)	0.02 [b]
NL2099	*rrf-3 aakg-5* RNAi	7	12.0 ± 0.40	-0.8 [a]	65 (67)	NS [b]
	N2 control RNAi	8	9.7 ± 0.25		96 (99)	
	N2 *aakg-4* RNAi	8	10.2 ± 0.22	+5.22 [a]	116 (122)	0.1370 [a]
	N2 *aakg-5* RNAi	8	10.7 ± 0.27	+10.61 [a]	98 (99)	0.0045 [a]
GA1071	*aakg-4* control RNAi	8	10.8 ± 0.26		96 (99)	0.0027 [a]
GA1071	*aakg-4; aakg-5* RNAi	8	12.2 ± 0.21	+12.37 [c]	97 (97)	0.0005 [c]
DR1567	*daf-2* control RNAi	8	21.5 ± 0.79		80 (85)	<0.0001[a]
DR1567	*daf-2 aakg-4* RNAi	8	19.7 ± 0.69	-8.43[b]	106 (111)	0.0013 [b]

DR1567	*daf-2 aakg-5* RNAi	8	16.9 ± 0.69	-21.24[b]	55 (67)	<0.0001[b]
	N2 control RNAi	9	9.9 ± 0.29		100 (100)	
	N2 *aakg-4* RNAi	9	9.9 ± 0.34	+0.68 [a]	64 (64)	NS [a]
	N2 *aakg-5* RNAi	9	10.0 ± 0.31	+1.72 [a]	100 (100)	NS [a]
GA1071	*aakg-4* control RNAi	9	10.4 ± 0.26		98 (99)	NS [a]
GA1071	*aakg-4; aakg-5* RNAi	9	10.6 ± 0.29	+2.46 [c]	100 (100)	NS [c]
DR1567	*daf-2* control RNAi	9	23.8 ± 0.71		85 (87)	<0.0001[a]
DR1567	*daf-2 aakg-4* RNAi	9	20.0 ± 0.57	-15.72 [b]	95 (95)	<0.0001[b]
DR1567	*daf-2 aakg-5* RNAi	9	20.2 ± 0.74	-14.92 [b]	90 (90)	0.0028 [b]
GA1072	*daf-2;aakg-4* control RNAi	9	18.2 ± 1.00		38 (38)	<0.0001[b]
GA1072	*daf-2;aakg-4 aakg-5* RNAi	9	19.7 ± 0.74	+7.95 [d]	80 (80)	NS [d]
DR1567	*daf-2* control RNAi	10	24.1 ± 0.73		105 (115)	-
DR1567	*daf-2 aakg-5* RNAi	10	18.5 ± 0.75	-23.40 [b]	99 (101)	<0.0001[b]
GA1072	*daf-2;aakg-4* control RNAi	10	18.9 ± 0.73	-21.65 [b]	71 (71)	<0.0001[b]
GA1072	*daf-2;aakg-4 aakg-5* RNAi	10	19.5 ± 0.67	+3.23 [d]	86 (95)	NS [d]

Table S8. *aakg-4* and *aakg-5* RNAi shorten *daf-2* lifespan.
