# Supplementary material for: DAF-16/FoxO Directly Regulates an Atypical AMP-Activated Protein Kinase Gamma Isoform to Mediate the Effects of Insulin/IGF-1 Signaling on Aging in Caenorhabditis elegans
Source: PLoS Genet. 2014 Feb 6;10(2):e1004109. doi: 10.1371/journal.pgen.1004109 (PMC3916255; doi:10.1371/journal.pgen.1004109)
Supplement: Table S9 — RNAi targeted to aakg-1, aakg-2 or aakg-3 does not consistently suppress daf-2 mutant longevity. N, number of worms (total worms scored including those censored). Lifespan assays all carried out at 25°C. The rrf-3 mutation sensitizes the animals to the effects of RNAi [52]. p, probability (determined by log rank test) of being the same as a rrf-3; daf-2 control RNAi or b rrf-3 Control RNAi. % differences in mean lifespan are compared to daf-2(m577). SEM: standard error of the mean. (PDF) [file pgen.1004109.s025.pdf]

Strain	Genotype and RNAi treatment	Trial	Mean lifespan ± SEM (days)	% Differences	N (total)	*p*
GA303	*rrf-3; daf-2* control RNAi	1	29.2 ± 0.98		73 (98)	-
GA303	*rrf-3; daf-2 aakg-1* RNAi	1	26.0 ± 0.71	-31	74 (84)	<0.0001[a]
GA303	*rrf-3; daf-2 aakg-2* RNAi	1	30.4 ± 1.21	+4.2	35 (43)	NS [a]
GA303	*rrf-3; daf-2 aakg-3* RNAi	1	28.2 ± 1.11	-3.4	35 (43)	NS [a]
NL2099	*rrf-3* control RNAi	2	14.8 ± 0.52		86 (99)	-
GA303	*rrf-3; daf-2* control RNAi	2	28.4 ± 1.08		86 (88)	<0.0001[b]
GA303	*rrf-3; daf-2 aakg-1* RNAi	2	28.7 ± 0.79	+0.8	103 (116)	NS [a]
GA303	*rrf-3; daf-2 aakg-2* RNAi	2	29.2 ± 1.07	+2.6	75 (83)	NS [a]
GA303	*rrf-3; daf-2 aakg-3* RNAi	2	32.3 ± 0.65	+13.8	104 (104)	NS [a]
NL2099	*rrf-3* control RNAi	3	13.7 ± 0.47		82 (82)	-
GA303	*rrf-3; daf-2* control RNAi	3	26.5 ± 0.83		106 (118)	<0.0001[b]
GA303	*rrf-3; daf-2 aakg-1* RNAi	3	24.9 ± 0.79	-6.2	129 (129)	NS [a]
GA303	*rrf-3; daf-2 aakg-2* RNAi	3	26.0 ± 0.90	-1.9	98 (98)	NS [a]
GA303	*rrf-3; daf-2 aakg-3* RNAi	3	31.3 ± 0.61	+18.1	125 (125)	<0.0001[a]
NL2099	*rrf-3* control RNAi	4	12.6 ± 0.34		119 (123)	-
GA303	*rrf-3; daf-2* control RNAi	4	26.6 ± 0.86		127 (127)	<0.0001[b]
GA303	*rrf-3; daf-2 aakg-1* RNAi	4	23.4 ± 1.09	-11.9	70 (71)	0.005 [a]
GA303	*rrf-3; daf-2 aakg-2* RNAi	4	25.9 ± 0.92	-2.5	109 (109)	NS
GA303	*rrf-3; daf-2 aakg-3* RNAi	4	24.8 ± 0.91	-6.8	85 (86)	NS

Table S9. RNAi targeted to *aakg-1, aakg-2* or *aakg-3* does not consistently suppress *daf-2* mutant longevity.
