# Supplementary material for: DAF-16/FoxO Directly Regulates an Atypical AMP-Activated Protein Kinase Gamma Isoform to Mediate the Effects of Insulin/IGF-1 Signaling on Aging in Caenorhabditis elegans
Source: PLoS Genet. 2014 Feb 6;10(2):e1004109. doi: 10.1371/journal.pgen.1004109 (PMC3916255; doi:10.1371/journal.pgen.1004109)
Supplement: Table S10 — Expression pattern of AMPK subunits. Y = expression observed in this tissue. Dark grey boxes highlight tissues where Paak-2::GFP, Paakb-1::GFP and Paakg-4::GFP expression was observed. (PDF) [file pgen.1004109.s026.pdf]

	Tissue	*Paak-2::GFP*	*Paakb-1::GFP*	*Paakg-4::GFP*	*Paakg-5::GFP*
**Head**	Excretory cell	Y			Y
	Pharyngeal neurons	Y	Y	Y	Y
	Pharyngeal epithelial cells	Y			Y
	Pharyngeal support cells				
	Pharyngeal ring neurons	Y	Y		Y
	Sensory neurons termini				Y
	Pharynx muscles		Y		
	Pharyngeal intestinal valve		Y		
	Ring ganglia			Y	
	Amphid sheath cells			Y	
	Amphid socket cells	Y		Y	
	Amphid neurons			Y	
	Body wall muscles (head)	Y			
**Mid-body**	Body wall muscles (mid)		Y	Y	
	Intestine (mid)	Y	Y	Y	Y
	Vulva muscles	Y	Y	Y	
	Uterine muscles		Y	Y	
	Vulva epithelium				Y
	Vulva neurons				Y
	Ventral cord processes/neurons			Y	Y
	Spermatheca				Y
	Epthelial seam cells				Y
	Female gonad sheath cells				Y
**Tail**	Body wall muscles (tail)		Y	Y	
	Intestine (posterior)	Y	Y	Y	Y
	Phasmid sheath cells	Y		Y	Y
	Phasmid socket cells	Y		Y	Y
	Phasmid neurons	Y		Y	
	Dorsal cord neuronal processes			Y	
	Rectal epithelial cells	Y		Y	Y
	Anal-depressor muscle			Y	
	Pre-anal ganglion rectal neurons			Y	Y
	Rectal gland	Y			
	Rectal intestinal valve		Y		Y
	Tail epithelia		Y		

Table S10. Expression pattern of AMPK subunits.
